# Genomic characterization of human papillomavirus-positive and -negative human squamous cell cancer cell lines

**DOI:** 10.18632/oncotarget.21174

**Published:** 2017-09-21

**Authors:** Nene N. Kalu, Tuhina Mazumdar, Shaohua Peng, Li Shen, Vaishnavi Sambandam, Xiayu Rao, Yuanxin Xi, Lerong Li, Yuan Qi, Frederico O. Gleber-Netto, Ameeta Patel, Jing Wang, Mitchell J. Frederick, Jeffrey N. Myers, Curtis R. Pickering, Faye M. Johnson

**Affiliations:** ^1^ Department of Thoracic/Head & Neck Medical Oncology, The University of Texas MD Anderson Cancer Center, Houston, Texas, USA; ^2^ Department of Bioinformatics and Computational Biology, The University of Texas MD Anderson Cancer Center, Houston, Texas, USA; ^3^ Department of Head and Neck Surgery, The University of Texas MD Anderson Cancer Center, Houston, Texas, USA; ^4^ The University of Texas Graduate School of Biomedical Sciences, Houston, Texas, USA; ^5^ Department of Otolaryngology, Baylor College of Medicine, Houston, Texas, USA; ^6^ Current/Present address: Lonza Viral Therapy, Houston, Texas, USA

**Keywords:** human papillomavirus, cervical cancer, head and neck squamous cell carcinoma, cell lines, mutation

## Abstract

Human cancer cell lines are the most frequently used preclinical models in the study of cancer biology and the development of therapeutics. Although anatomically diverse, human papillomavirus (HPV)-driven cancers have a common etiology and similar mutations that overlap with but are distinct from those found in HPV-negative cancers. Building on prior studies that have characterized subsets of head and neck squamous cell carcinoma (HNSCC) and cervical squamous cell carcinoma (CESC) cell lines separately, we performed genomic, viral gene expression, and viral integration analyses on 74 cell lines that include all readily-available HPV-positive (9 HNSCC, 8 CESC) and CESC (8 HPV-positive, 2 HPV-negative) cell lines and 55 HPV-negative HNSCC cell lines. We used over 700 human tumors for comparison. Mutation patterns in the cell lines were similar to those of human tumors. We confirmed HPV viral protein and mRNA expression in the HPV-positive cell lines. We found HPV types in three CESC cell lines that are distinct from those previously reported. We found that cell lines and tumors had similar patterns of viral gene expression; there were few sites of recurrent HPV integration. As seen in tumors, HPV integration did appear to alter host gene expression in cell lines. The HPV-positive cell lines had higher levels of p16 and lower levels of Rb protein expression than did the HPV-negative lines. Although the number of HPV-positive cell lines is limited, our results suggest that these cell lines represent suitable models for studying HNSCC and CESC, both of which are common and lethal.

## INTRODUCTION

Head and neck squamous cell carcinoma (HNSCC) is the seventh most common cancer worldwide, with over 600,000 cases annually [1]. More than 50,000 new cases occur each year in the United States alone [2]. The age-adjusted incidence of oropharyngeal cancer has been increasing at an alarming rate of 5% per year over the past decade in the United States [3] and at a similar pace in other industrialized countries [4]. No significant improvements in the overall survival of patients with advanced disease have been made in about 3 decades. One of the risk factors associated with the marked increase in HNSCC cases is infection with high-risk types of human papillomavirus (HPV), which is associated with 71% of oropharyngeal cancer cases [5]. HPV was first identified as a causative agent for cervical squamous cell carcinoma (CESC) about 40 years ago [6]. Despite advances in early screening, HPV-associated CESCs are still the number one cause of death in women worldwide, with nearly 90% of the mortalities occurring in the developing world [5, 7–9]. It is estimated that by 2020, HPV-positive oropharyngeal cancer cases will outnumber HPV-driven CESC cases in the United States [3]. Additionally, HPV has been implicated in squamous cell cancers of the anogenital region, including penile, vulvar, and anal cancers [8, 9].

Although HPV-associated and non-HPV-associated HNSCCs display clinical, epidemiologic, and molecular differences, the therapeutic regimens remain the same for both etiologies [10]. The discovery of new therapeutic interventions for HNSCC requires conducting preclinical studies, which rely heavily on human tumor cell lines. Several reports have identified actionable molecular targets by investigating the genomic characteristics of HNSCC cell lines and tumors [11–13]. Recent mutational and transcriptomic analyses of up to 22 HNSCC cell lines have provided valuable data on the characteristics of HNSCCs [11, 14, 15]. The results of short tandem repeat (STR) profiling of 61 unique HNSCC cell lines have also been published [16]. While researchers worldwide frequently use these cell lines and some of their characteristics have been published in a piecemeal fashion, the genomic characteristics of the available HNSCC and CESC cell lines have not been thoroughly investigated and compared in a single study. To complement and extend prior studies, we performed genomic and proteomic analyses of 55 HPV-negative and 9 HPV-positive HNSCC cell lines. These cell lines represent the vast majority of the preclinical models currently used worldwide for *in vitro* and *in vivo* biomarker and targeted therapy identification for HNSCC. In order to tease out the genomic alterations attributable to HPV, we also included CESC cell lines, which are predominantly HPV-positive, in our analyses. The purpose of this study was to determine the genomic profiles—including mutations, HPV integration, and viral gene expression—of a large number of frequently used HPV-negative HNSCC cell lines and a majority of the HPV-positive tumor cell lines. Through these analyses, we found similar incidences of common mutations and genomic alterations in targetable genes as observed in previously performed preclinical studies as well as in the clinical samples curated by the cancer genome atlas (TCGA), suggesting that these cell lines are suitable candidates for studying HNSCC and CESC.

## RESULTS

### Cell line selection and authentication with STR profiling

Despite the recent increase in the incidence of HPV-positive HNSCC and the high global prevalence of CESC, only 17 HPV-positive cell lines are widely used in research (9 HNSCC and 8 CESC cell lines). We characterized these cell lines and, for comparison, 2 commonly used HPV-negative CESC cell lines and 55 HPV-negative HNSCC cell lines, for a total of 74 cell lines (Supplementary Table 1). Like the patient tumors in TCGA, HPV-positive HNSCC cell lines are predominantly HPV16-positive, and CESC cell lines are predominantly HPV16- and HPV18-positive [17]. We performed STR analysis to confirm cell line identity and conducted whole exome sequencing, mRNA sequencing (RNASeq), and reverse phase protein array (RPPA) analysis for genomic and proteomic characterization of the cell lines. The STR profiles of many of these cell lines have been previously reported [16]; however, we report here for the first time STR profiles of several HPV-positive HNSCC cell lines (Supplementary Table 2). Before conducting the genomic and proteomic analyses, we determined the doubling times of the HPV-positive HNSCC and CESC cell lines. Characterization of the doubling times of the HPV-negative HNSCC cell lines had been previously performed [16]. Therefore, we focused on the HPV-positive cell lines and all the CESC cell lines (Supplementary Table 3). Overall, the HPV-positive HNSCC cell lines had longer doubling times than did the CESC cell lines (mean: 69 hours vs. 51 hours), but the difference was not statistically significant (*t* test *p* = 0.12).

### HNSCC and CESC cell line mutation patterns resemble those of patient tumors

**Figure 1:**
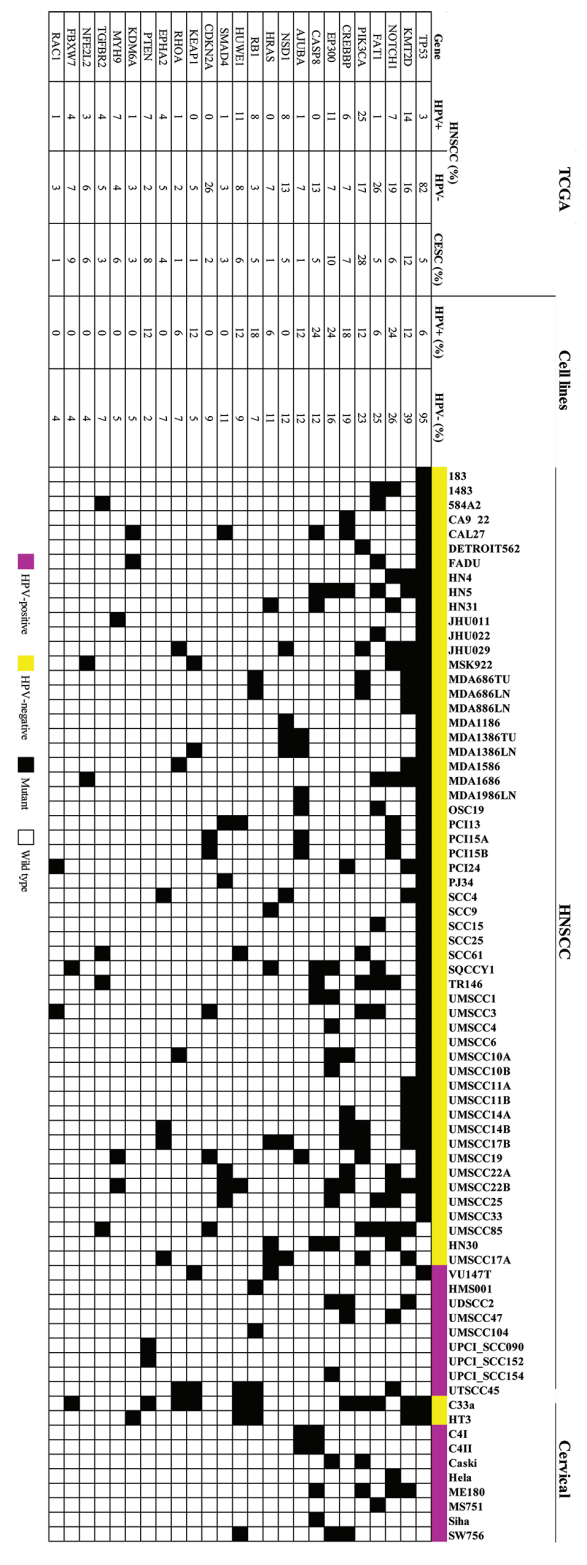
HNSCC and CESC cell lines have mutation frequencies similar to those in patient tumors All HNSCC and CESC cell lines were subjected to whole exome sequencing. The 25 most frequently mutated genes are listed by cell line. For comparison, mutation frequencies of 431 HPV-negative HNSCC, 79 HPV-positive HNSCC, and 194 CESC human tumors were compiled from TCGA.

Whole exome sequence analysis revealed distinct genetic mutations in HPV-positive and HPV-negative cell lines (Figure 1). *TP53* mutations were detected in 98% of the HPV-negative HNSCC cell lines and 82% of the 431 HPV-negative HNSCC tumors from TCGA. In contrast, *TP53* mutations were far less common (5%) in the 194 TCGA CESC tumors, which are predominantly HPV-positive and *TP53* was mutated in only the 2 HPV-negative CESC cell lines [18]. Only 1 HPV-positive HNSCC cell line (94VU147T) harbored a *TP53* mutation, as previously reported [11]. The second most frequently mutated gene was *KMT2D*, which encodes the protein lysine methyltransferase 2D and was mutated in 31% of the cell lines – predominantly in the HPV negative lines (39%). *KMT2D* was also mutated in tumors, but at a lower frequency (12–16%). The most frequently mutated genes in HPV-positive cell lines were *NOTCH1, EP300, and CASP8* (24%), which were also mutated in 7, 11, and 0% of HPV-positive HNSCC and 6, 10, and 5% of CESC tumors from TCGA respectively. In contrast to HNSCC tumors, no HPV-positive HNSCC cell lines had *PIK3CA* mutations, although 2 (25%) HPV-positive CESC cell lines did. *CDKN2A* (9%), *FAT1* (23%), *SMAD4* (11%), and *CASP8* (13%) mutations were only present in HPV-negative HNSCC cell lines, which was consistent with the HNSCC TCGA data, where each of these mutations occurred in only 1 HPV-positive HNSCC patient tumor. *NOTCH1* mutations were present in 15 (28%) HPV-negative HNSCC cell lines, 2 (22%) HPV-positive HNSCC lines, and 2 (25%) HPV-positive CESC cell lines.

### Viral gene expression patterns

**Figure 2:**
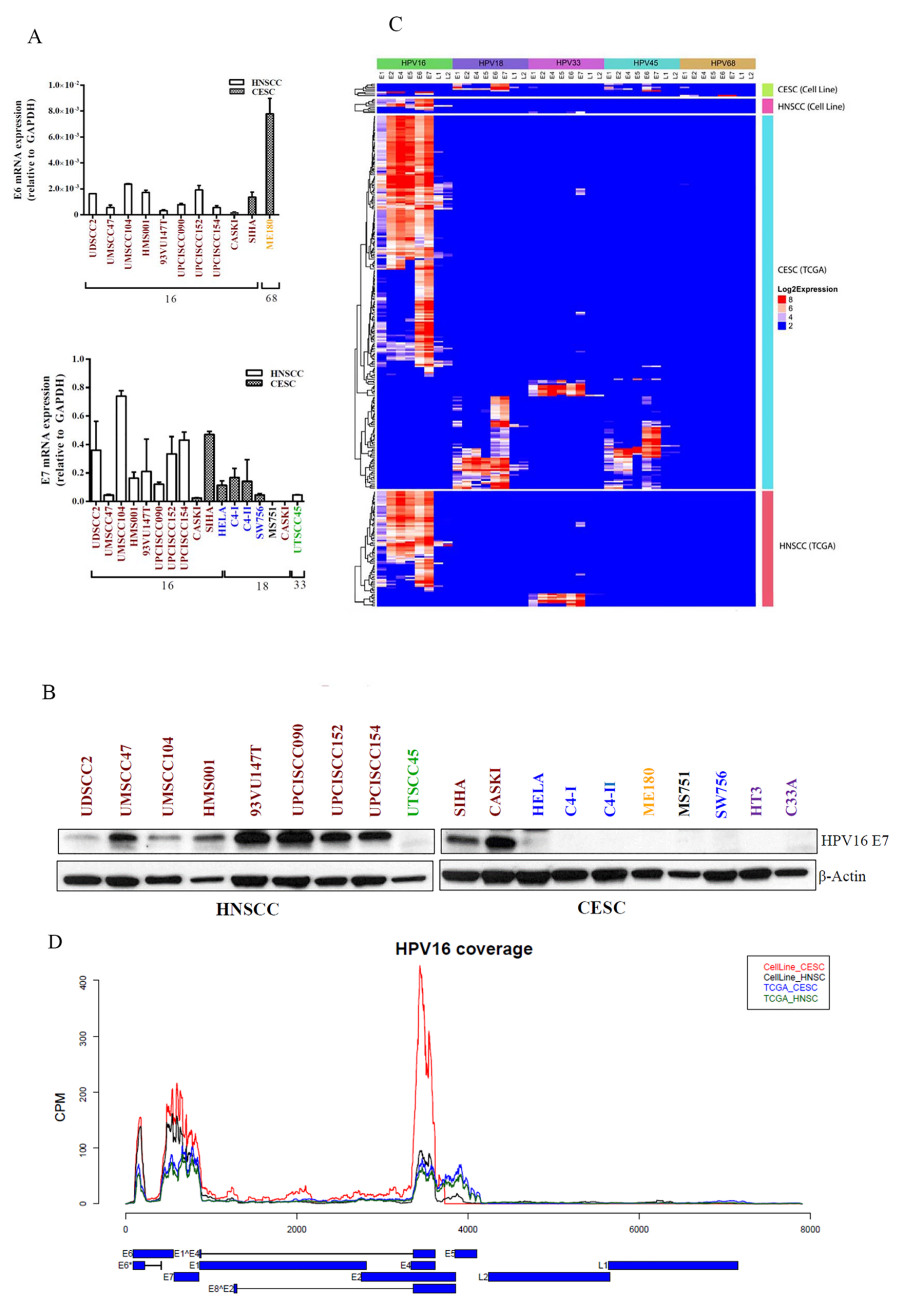
HPV viral gene expression in HNSCC and CESC cell lines and TCGA tumors mRNA **(A)** and protein **(B)** were extracted from HPV-positive cell lines and subjected to qPCR and immunoblotting, respectively, for the viral oncogenes E6 and E7. GAPDH and β-actin were used as controls, respectively. Text color indicates HPV viral type: red, HPV16; blue, HPV18; gold, HPV68; black, HPV45; green, HPV33. HPV gene expression patterns in all HPV-positive samples from CESC and HNSCC cell lines (total 17) and CESC and HNSCC TCGA patient samples (total 301) for 5 HPV types (HPV16, HPV18, HPV33, HPV45, and HPV68) **(C)** or just HPV16-positive cell lines and TGCA samples **(D)**. Reads coverage for HPV16-positive cell lines and TCGA samples. Expression levels are represented as reads counts per million reads (CPM). The HPV16 gene structures are presented in the bottom panel, with blue blocks represent exons and black lines represent introns.

The roles of HPV oncoproteins E6 and E7 that promote apoptosis and cell cycle progression by respectively targeting p53 and Rb proteins for degradation are well established [19, 20]. To determine mRNA levels of the HPV oncogenes E6 and E7, we performed real time, quantitative reverse transcription polymerase chain reaction (RT-PCR) amplification using primers targeting these genes [19, 20]. We detected the presence of HPV16 E6 and E7 mRNA and/or protein in all 10 HPV16-positive cell lines (Figure 2A). We also detected HPV33 E7 mRNA in UTSCC45 cells and HPV18 E7 mRNA in 4 HPV18-positive cell lines. The HPV type for 3 cell lines differed from that reported in the literature. The HPV type for ME180 cells is variably reported as HPV18, HPV39, or HPV68 [21–23]. We detected neither E6 nor E7 from HPV18 or HPV39 in ME180 cells using RT-PCR. However, we did detect HPV68 E6 expression by RT-PCR and HPV68 E1, E2, E6, and E7 expression by RNASeq in these cells (Supplementary Figure 1). Furthermore, we detected no integrated HPV sequences in ME180 cells (integration described below). In MS751 cells, we did not detect HPV18 E7 by RT-PCR but did detect integration of HPV45 (described below). Finally, in CaSki cells, we detected HPV16 but not HPV18 [24]. Detection of E6 and E7 proteins was limited by a lack of reliable antibodies, but we were able to detect HPV16 E7 protein in all 10 HPV16-positive cell lines using Western blotting (Figure 2B).

We used transcriptome analysis (RNASeq) to identify the HPV genes expressed (Supplementary Figures 1-2) and integrated (Table 1) in the HPV-positive cell lines and TCGA tumors and detected E1, E2, E4, E5, E6, E7, L1, and L2 expression at variable levels in tumors and cell lines. The expression level of E5 was lower in cell lines than in tumors. Otherwise, viral gene expression levels were similar in cell lines and tumors, although the low number of cell lines and wide variability of expression levels precluded a formal comparison (Figures 2C–2D; Supplementary Figures 1 and 2). As previously described [25, 26], the predominant form of E6 in both cell lines and tumors was E6*.

**Table 1:** HPV genes found to be integrated in cell lines and TCGA tumors

Sample type	Cancer type	HPV types	HPV genes
Cell Line	CESC	16, 18, 45	E1, E2, E4, E6, E7, L1
Cell Line	HNSCC	16, 33	E1, E2, E4, E5, E6, E7, L1, L2
TCGA	CESC	16, 18, 33, 35, 45	E1, E2, E4, E5, E6, E7, L1, L2
TCGA	HNSCC	16, 18, 33, 35, 56	E1, E2, E4, E5, E6, E7, L1, L2

HPV, human papillomavirus; TCGA, The Cancer Genome Atlas; CESC, cervical squamous cell carcinoma; HNSCC, head and neck squamous cell carcinoma

### Detection of HPV integration sites

**Figure 3:**
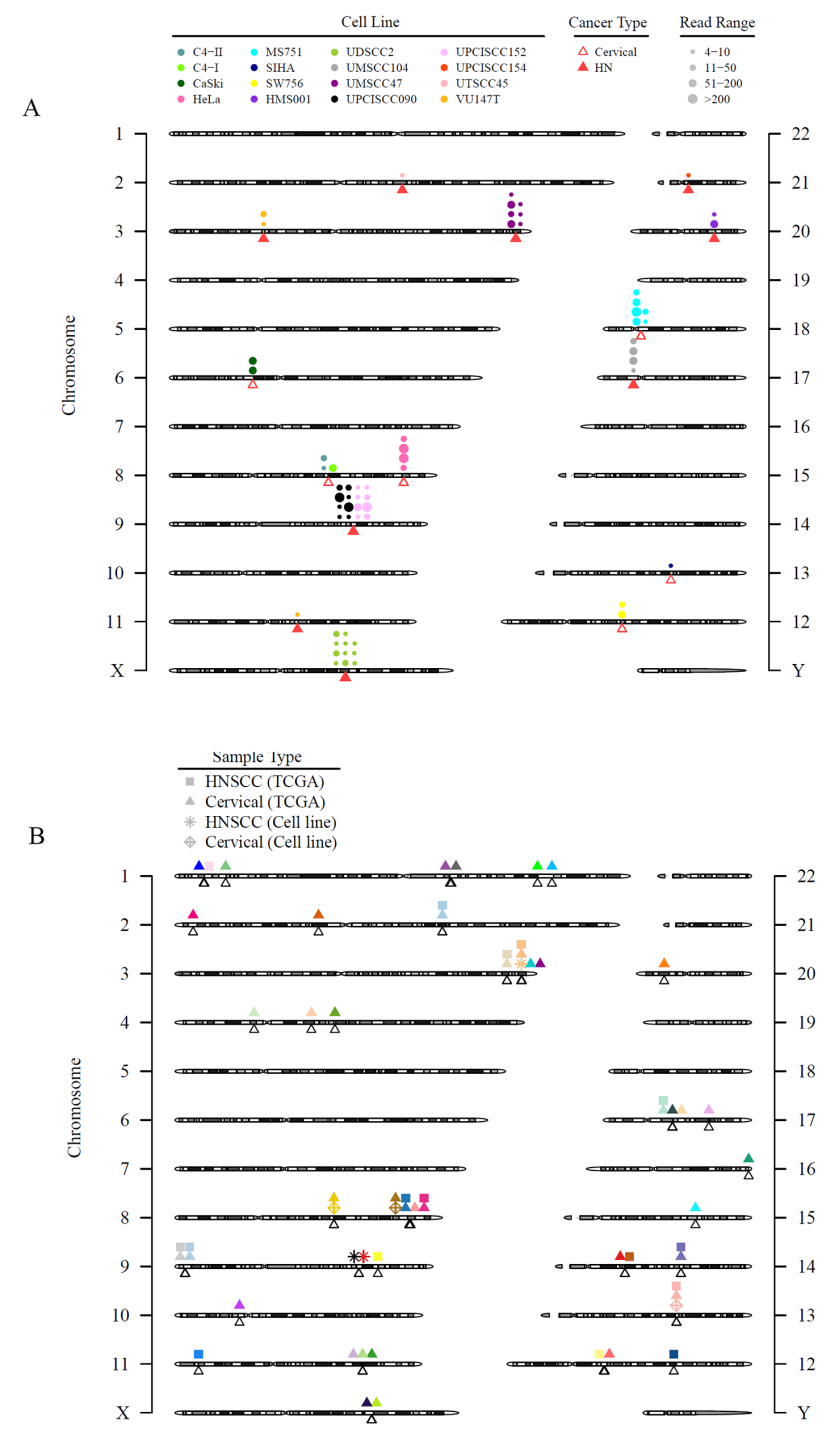
HPV integration sites in HNSCC and CESC cell lines and tissues are diverse with few areas of recurrent integration Viral integration sites for all HPV-positive cell lines alone **(A)** and combined with the TCGA HNSCC and CESC tumors **(B)**. (A) Triangles point to the exact integration locations and may overlap. Each circle represents an integration event; circles that are close to each other may be shifted for better visualization. The size of the circles represents discordant read pairs in different ranges. A read pair was reported as discordant if the paired-end reads were uniquely mapped with one end to a human chromosome and the other to the virus chromosome. Discordant read pairs are evidence of HPV integration. Larger circles indicate that more discordant read pairs were mapped to that integration site and, therefore, provide stronger evidence for integration. (B) Unfilled triangles point to the exact integration event and may overlap. The shapes of the symbols refer to different sample types or sources. Each color represents a gene, but the location of the symbols may be shifted slightly for better visualization. Only genes with HPV integration in 2 or more patient tumor samples in TCGA or cell lines were plotted.

We investigated HPV integration in the cell lines and patient tumors from the TCGA using whole exome sequencing data. Similar to several previous reports summarizing studies of HPV integration sites, we confirmed HPV integration sites in HeLa, SiHa, CaSki, UPCISCC090, 93VU147T, HMS001, UDSCC2, and UMSCC47 cell lines by using RNASeq [12, 27]. Furthermore, we newly identified HPV integration sites in UPCISCC152, UPCISCC154, UMSCC104, UTSCC45, C4-I, C4-II, SW756, and MS751 cell lines (Supplementary Table 4, Figure 3A). While we confirmed the presence of HPV68 in ME180 by RT-PCR, no HPV integration sites were present in this CESC cell line.

Most of the observed HPV integration sites in the HNSCC and CESC cell lines were not recurrent, as they were identified in only 1 cell line (Figure 3A). Cell lines established from the same patients (UPCISCC090/UPCISCC152 and C4-I/C4-II) showed integration events at the same location. Combined data from 522 HNSCC tumors (72 HPV-positive) and 306 CESC tumors (276 HPV-positive) in the TCGA and our cell lines demonstrated 1923 integration events (Figure 3B; Supplementary Figure 3). Recurrent integrations—those that affected 5 or more cases—occurred in only 7 genes encoding 4 transcription factors, *KLF12* (n = 15), *MYC* (n = 7), *POU5F1B* (n = 7), and *TP63* (n = 6); a candidate oncogene, *PVT-1* (n = 11); a DNA repair protein, *RAD51B* (n = 7); and the receptor tyrosine kinase *ERBB2* (n = 6) (Table 2). Twenty-five genes had recurrent integrations in 2 or more samples, including programmed cell death-ligand 1 (*PD-L1)* (n = 3) (Figure 3B and Supplementary Table 5). The recurrent integrations that were detected in both cell lines and patient samples were in *KLF12* (SiHa cells), *POU5F1B* (HeLa cells), and *TP63* (UMSCC47 cells).

**Table 2:** Genes in which HPV was integrated in at least 5 TCGA tumors or HPV positive cell lines

Gene name	Protein name	Number of samples with integration	Chromosome	Function	Role in cancer
*KLF12*	Krueppel-like factor 12	15	13	Represses AP-2 alpha gene expression	Regulates gene expression during vertebrate development and carcinogenesis
*PVT1*	PVT-1 oncogene	11	8	Long noncoding RNA locus identified as a candidate oncogene	Implicated in breast and ovarian cancer, AML, and Hodgkin lymphoma
*POU5F1B*	POU class 5 homeobox 1B	7	8	Homeobox 1 transcription factor	Induces upregulation of growth factors and promote proliferation
*RAD51B*	RAD51 paralog B	7	14	Homologous recombination repair pathway	Overexpression causes cell cycle G1 delay and apoptosis
*MYC*	c-Myc	7	8	Activates the transcription of growth-related genes	Oncogene that regulates the cancer epigenome and transcriptome
*TP63*	Tumor protein 63	6	3	Member of the p53 family of transcription factors	Isoforms involved in adult stem/progenitor cell regulation
*ERBB2*	Erb-b2 receptor tyrosine kinase 2	6	17	Binds to ligand-bound EGF receptor family members, stabilizes ligand binding, and enhances downstream signaling pathways	Amplification and/or overexpression is reported in numerous cancers, including breast and ovarian tumors

### HPV integration alters host gene expression

**Figure 4:**
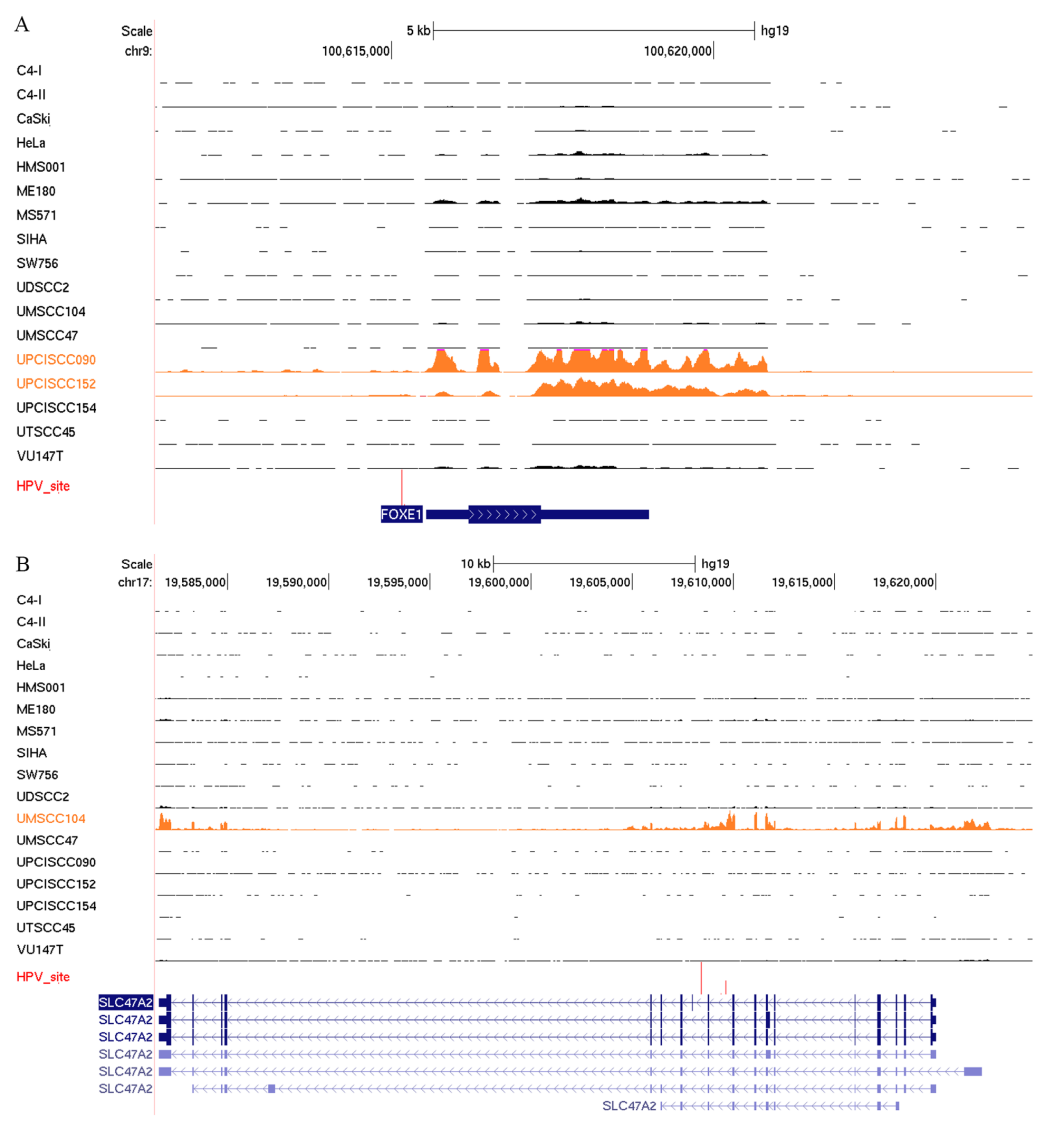
HPV integration alters host gene expression RNASeq reads occupancy profile for *FOXE1* **(A)** and *SLC47A2* **(B)** genes in CESC and HNSCC cell lines. Text and data for the cell lines with the HPV integration sites (red vertical lines) detected near these genes appear in orange.

Several reports have demonstrated the enrichment of HPV integration sites within or near cellular genes [12, 27]. We evaluated the effects of HPV integration on cellular gene expression levels in the HPV-positive cell lines. Genes that were altered by HPV integration were more highly expressed than were the same genes that were unaffected by HPV integration in other HPV-positive cell lines. For instance, we confirmed previously reported altered expression of *FOXE1* in UPCISCC090 and UPCISCC152 cell lines and of *SLC47A2* in UMSCC104 cells [12] (Figure 4). We also identified decreased expression of *NR1P1*, a nuclear receptor protein, and *USP25*, a ubiquitin-specific peptidase, in UPSCISCC154 cells (Supplementary Figure 4).

### Differential gene and protein expression of HNSCC and cervical cancer cell lines

We compared global gene expression patterns in 74 HNSCC and CESC cell lines. The majority of the CESC cell lines clustered together, with more variable results for the HPV-positive HNSCC cell lines. Cell lines from the same patients also clustered together (Supplementary Figure 5). We performed RPPA proteomic profiling on all the HPV-positive HNSCC cell lines and a comparison group of HPV-negative HNSCC cell lines in the same batch to eliminate batch effects. The samples were probed with 305 antibodies targeting total and/or phosphorylated proteins found in oncogenic signaling pathways (Supplementary Table 6). Two sample t-test was applied to identify differentially expressed proteins. The resulting p values were fitted by a beta-uniform mixture model [27] in order to correct for multiple hypothesis testing and to estimate false discovery rate (FDR). We found 23 proteins that were differentially expressed at a FDR of 0.2 (Supplementary Table 7) including p16 that results from Rb inactivation and is used as a clinical marker for HPV expression [28]. As anticipated, HPV-positive cell lines had significantly higher expression levels of p16 and lower expression of phosphorylated Rb (pRb) than did HPV-negative cell lines (Supplementary Figure 6).

## DISCUSSION

In this study, we characterized mutations, viral gene expression, and viral integration in all the readily available HPV-positive HNSCC cell lines, 10 CESC cell lines, 55 HPV-negative HNSCC cell lines, and over 700 patient tumors from TCGA. Using whole exome sequencing, we identified mutations in the cell lines that were similar in frequency to those found in patient tumors. As expected, HPV-positive cell lines expressed higher levels of p16 and lower levels of pRb than did HPV-negative cell lines. With the exception of cell lines derived from the same patients, each cell line had a different HPV integration site. Similarly, patient tumors had few recurrent viral integration sites. Also, as previously demonstrated for tumors, HPV altered the expression of genes near the integration sites.

Several other investigators have characterized HNSCC and CESC cell lines [4, 11, 15, 16, 27, 29, 30]. Those studies have served as resources for the cancer research community, which relies on these established models. Our research builds on and integrates those studies, as we have characterized and compiled the most comprehensive set of HPV-positive HNSCC and CESC cell lines, with a large number of HPV-negative HNSCC cell lines for comparison. We confirmed the HPV positivity of all the HPV-positive cell lines but found that the type of HPV differed from that reported in the literature for 3 of the CESC cell lines. The differences between our results and those reported in the literature are likely attributable to the different HPV detection methods we used, as exemplified by the results for ME180 cells. Before PCR came into wide use, HPV18 DNA was detected in ME180 cells by DNA hybridization, but no HPV18 RNA was detected in these cells [21]. However, hybridization conducted under more stringent conditions failed to detect HPV18, though sequencing identified HPV39 in ME180 cells [22]. Later, Longuet et. al. [23] cloned and amplified HPV68 from ME180 cells.

The mutation profiles of the 74 cell lines included in this study resembled those found in similar human tumors. We identified *TP53* mutations almost exclusively in HPV-negative cell lines. We also identified frequent mutations in *CASP8*, *NOTCH1*, *FAT1*, and *PIK3CA*. One noteworthy finding in the HPV-positive HNSCC cell lines was the absence of *PIK3CA* mutations. However, HPV-negative HNSCC and HPV-positive CESC cell lines had *PIK3CA* mutations at rates similar to those found in human tumors, suggesting that these mutations are not selected against during cell line creation.

We confirmed published HPV integration sites for 8 cell lines and found identical integration sites in cell lines that originated from the same patients, demonstrating that our technique is reproducible. We also newly defined HPV integration sites in 8 additional cell lines. As in HPV-positive CESC and HNSCC tumors [12, 27], we found very few recurrent integration sites in our cell lines, even though the majority of HPV-positive cell lines had viral integration. Until recently, HPV integration events were thought to be random, and the main function of HPV integration was believed to be stabilization of viral oncogene transcription [31]. However, more recent reports using next-generation sequencing showed that integration events frequently occur (> 80% of the time) within or near known oncogenes, tumor suppressor genes, or DNA repair genes [12, 27, 32]. These findings support the idea of nonrandom integration, in which cells containing HPV integration breakpoints in specific genes are selected for and may play a role in cancer progression. Consistent with this hypothesis, we observed altered host gene expression at or near HPV integration sites, similar to published findings in HPV-positive tumors [12, 32, 33].

The levels of HPV E6 and E7 expression as measured by RT-PCR, Western blot, and RNASeq varied significantly across cell lines. Similarly, E6 and E7 mRNA expression (as determined by RNASeq) varied significantly between tumors from the TCGA. Although more than 3 decades of research on HPV-associated cancers has solidified the importance of viral oncogenes E6 and E7 [20, 34], their expression is not sufficient for tumor development *in vivo* [35, 36]. In addition to the oncogenic activities of E6 and E7, other events are required for full transformation to cancer. Likewise, inhibition or knockdown of E6 and E7 has varied effects in HPV-positive cell lines [37–39] which suggests that HPV-positive cancers have variable dependence on E6 and E7, leading to heterogeneity in the expression levels of these oncogenes.

One limitation of our study is that we identified HPV integration sites using whole exome sequencing, which is limited to exomes and prevents the detection of HPV integration events that do not occur in protein-coding regions of the genome. Whole genome sequencing may have identified additional sites of integration but was not performed because of its cost. Another limitation was the small number of HPV-positive cell lines that were available for study. The large disparity between the number of available HPV-positive and HPV-negative cell lines makes it difficult to perform statistical analyses that may reveal differences in gene and protein expression levels. Another limitation is a lack of HPV-positive oropharynx cell lines.

In summary, in this panel of 74 human HNSCC and CESC cell lines, the frequency and patterns of mutations resembled those found in human tumors. In the HPV-positive cell lines, viral gene expression and integration largely mirrored that found in human tumors. These results suggest that these widely used HNSCC and CESC cell lines are suitable models for studying HNSCC and CESC and may be useful in research that could lead to new therapies while emphasizing the need for the development of more HPV-positive models. Our analyses and characterization of these cell lines will serve as a valuable resource for the cancer research community.

## MATERIALS AND METHODS

### Cell lines and STR profiling

A panel of 74 HNSCC and CESC cell lines (Supplementary Table 1) were maintained in their respective growth media (Supplementary Table 3 and [16]). The UDSCC2 cell line was a kind gift from J. Silvio Gutkind (University of California, San Diego), 93VU147T from Josephine Dorsman (Vrije Universiteit Medical Center), and UTSCC45 from Reidar Grenman (University of Turku) [12, 30, 40–44]. The HPV-positive HNSCC cell lines included 2 cell lines established at the University of Michigan, UMSCC47 and UMSCC104 [40, 41]. Three HNSCC cell lines (UPCISCC090, UPCISCC152, and UPCISCC154) were established at the University of Pittsburgh [30, 42]. The HNSCC cell lines 93VU147T, UTSCC45, HMS001, and UDSCC2 were established at various institutions [12, 43–45]. The 8 HPV-positive CESC lines (HeLa, CaSki, SiHa, MS751, ME180, SW756, C4-I, and C4-II) were obtained from ATCC (Manassas, VA) [46]. Cell lines were validated by STR DNA fingerprinting using the Promega 16 High Sensitivity STR Kit (Catalog # DC2100; Promega, Madison, WI). The STR profiles were compared to online search databases (DSMZ/ATCC/JCRB/RIKEN) of 2455 known profiles and to the MD Anderson Characterized Cell Line Core database of 2556 known profiles. The STR profiles either matched known DNA fingerprints or were unique (Supplementary Table 2). All the cell lines were determined to be mycoplasma-free at the time of analysis using the MycoAlert mycoplasma detection kit (Lonza, Basel, Switzerland). The cell lines were maintained for no longer than 20 passages after recovery from frozen stocks.

### Growth curve analysis

Growth curve analyses were performed on cells prior to the tenth passage after thawing. The cells were passaged during log phase growth before they reached confluence. Cells were trypsinized with 0.25% trypsin/ethylenediaminetetraacetic acid and allowed to detach for 2 to 3 minutes at 37°C. Following trypsin inactivation with serum-containing media, the cells were counted and serially diluted from 2500 cells/mL to 40,000 cells/mL. The cells were then seeded in a 384-well plate (50 μL/well and 36 wells per dilution). The plated cells were allowed to attach at room temperature for 45 minutes and then placed in the cell culture incubator overnight. Each day for 4 days, 7 wells from each cell dilution were fixed, stained with 4′,6-diamidino-2-phenylindole, and counted. The doubling times were calculated using the formula ${\text{T}}_{\text{d}}=3*\left(\text{LOG}\left[2\right]/\text{LOG}\left[\text{cell}\;\text{number}\;\text{at}\;96\;\text{h}/\text{cell}\;\text{number}\;\text{at}\;24\;\text{h}\right]\right)$.

### DNA and RNA isolation

For each cell line, genomic DNA was extracted from 1 to 2 million cells using the ArchivePure DNA cell/tissue kit (5Prime, South San Francisco CA) following the manufacturer’s instructions. The extracted DNA was stored in nuclease-free water (Ambion, Austin, TX). For each cell line, RNA was isolated from 5 million cells using the RNeasy Plus Kit (Catalog #73404; Qiagen, Germantown, MD) according to the manufacturer’s protocol and eluted into ribonuclease-free water.

### cDNA synthesis and real-time PCR for HPV mRNA and genomic DNA quantitation

cDNA was synthesized from 1 μg of RNA using the iScript cDNA Synthesis Kit (Catalog #170-8890; Bio-Rad, Hercules, CA) according to the manufacturer’s protocol. The synthesized cDNA was used as a template to determine the HPV E6 and E7 gene expression levels for HPV types 16, 18, 33, 45, and 68. To determine relative HPV viral gene expression levels, genomic DNA was isolated, and HPV E6, E7, and GAPDH primers were used for real-time, quantitative PCR (Supplementary Table 8). Each 20-μL RT-PCR reaction contained 1× SsoFast SYBR Green SuperMix (Bio-Rad), a 100-nM concentration of each primer, and 25 ng of DNA. Thermocycling conditions were 98°C for 2 minutes for 1 cycle, 98°C for 30 seconds, and 60°C for 5 seconds for 40 cycles.

### Immunoblotting (western blotting)

Cells were seeded at a density of 0.5 × 10^6^/mL in 10-cm dishes. The following day, cells were harvested and lysed using cell lysis buffer (Cell Signaling Technology, Danvers, MA) supplemented with a protease inhibitor cocktail. Cell lysates were incubated for 15 minutes on ice and then centrifuged for 5 minutes at 14,000 rpm. The collected supernatants were stored at −80°C. Protein concentrations were determined using a bicinchoninic acid assay (Catalog #23252; Thermo Scientific, Rockford, IL). Ten micrograms of total protein was separated by sodium dodecyl sulfate polyacrylamide gel electrophoresis, transferred onto a nitrocellulose membrane, and detected using ECL detection reagents (Catalog #RPN2232; GE Healthcare, Pittsburgh, PA). The nitrocellulose membranes were blocked for 1 hour using blocking buffer (5% milk, 1× phosphate-buffered saline, 0.1% Tween 20). The cells were incubated with anti-HPV16 E7 (Cervimax, Vienna, Austria) (1:500) overnight at 4°C. Protein expression was normalized using β-actin (Catalog #A1978; Sigma-Aldrich, St. Louis, MO).

### RNA sequencing

RNA was isolated from the cell lines using the RNeasy Plus kit (Qiagen). Illumina-compatible libraries were prepared using a TruSeq Stranded Total RNA Sample Prep Kit (Illumina, Inc., San Diego, CA). Briefly, 500 ng of DNase I-treated total RNA was depleted of ribosomal RNA using biotinylated, target-specific oligos. Following purification, the RNA was fragmented using divalent cations, and first-strand cDNA synthesis was carried out using random primers. Following second-strand synthesis, the ends of the resulting double-stranded cDNA fragments were repaired, 5′-phosphorylated, and 3′-A tailed. Illumina-specific Y-shaped indexed adapters were ligated. The products were then purified and enriched using PCR to create the final cDNA library. The libraries were quantified using qPCR (Kapa Biosystems, Wilmington, MA), assessed for size distribution using an Agilent Bioanalyzer (Agilent Technologies, Santa Clara, CA), and then multiplexed and sequenced on an Illumina HiSeq3000 sequencer using the 75-bp paired-end format. After sequencing, BCL files were converted to “.Fastq.gz” files, and individual sample libraries were de-multiplexed using CASAVA 1.8.2 software (Illumina) with no mismatches.

### Whole exome sequencing and mutation calling

NimbleGen SeqCap EZ libraries (Roche-NimbleGen, Madison, WI) were prepared per the manufacturer’s protocol. Briefly, indexed libraries were prepared from 0.5–1.0 μg of sheared and RNase-treated genomic DNA using the KAPA HTP Library Preparation Kit (Kapa Biosystems). The indexed libraries were amplified by 7 cycles of ligation-mediated PCR. Following amplification and reaction cleanup using Agencourt AMpure XP beads (Beckman Coulter, Brea, CA), the libraries were quantified fluorometrically using the Qubit dsDNA HS Assay (ThermoFisher, Waltham, MA) and assessed for size distribution using the Fragment Analyzer (Advanced Analytical, Ankeny, IA). Library concentrations were normalized, and the libraries were pooled at 250 ng/library with 4 to 6 libraries/pool. Each multiplexed library pool was hybridized to a probe pool from the SeqCap EZ Human Exome Enrichment Kit v3.0 (Roche-NimbleGen). Following capture, the exome-enriched libraries were amplified using 6 cycles of PCR and then purified using Agencourt AMpure XP beads. The libraries were then quantified fluorometrically using the Qubit dsDNA HS Assay and assessed for size distribution using the Fragment Analyzer. Exome enrichment efficiency was determined using qPCR. Sequencing was performed on a HiSeq4000 Sequencer (Illumina) using the 75-nt paired-end format, with 1 pool (4–6 libraries) sequenced per lane. Following sequencing, BCL files were converted to “.Fastq.” files, and individual sample libraries were de-multiplexed using bcl2fastq2 conversion software version 2.17.1.14 (Illumina).

The paired-end reads were aligned to human reference genome hg19 using BWA [47]. SAMtools flagstat was used to check the mapping quality [48]. Duplicate reads were removed using the Picard tool of MarkDuplicates (http://broadinstitute.github.io/picard/). Then, the Genome Analysis Toolkit indel realignment, base quality score recalibration, and SNP and INDEL discovery tools were applied for variant calling [49–51]. The variants were annotated using ANNOVAR with corresponding databases for human hg19 [52].

To account for the lack of matched normal DNA for established cancer cell lines, we employed several levels of variant filtering, as previously reported, to perform somatic mutation calls [11, 53]. The mutation filter removed any noncoding or silent sequences. The filtering steps also removed mutations found with high frequency in the 1000 Genomes Project and ESP6500 databases [54]. Next, we removed mutations found in 3 or more unique patients and rescued any splice site mutations, frameshift indels, stopgain mutations, and stoploss mutations that may have been removed. We also rescued CLINVAR “pathogenic” mutations and COSMIC mutations that were present in more than 4 cases, as we described previously [55].

### Reverse phase protein array

Cell lysates were serially diluted twofold for 5 dilutions (from undiluted to 1:16 dilution) and arrayed on nitrocellulose-coated slides in an 11 × 11 format, as we described previously [56, 57]. We stained the RPPA slides with 305 unique antibodies (Supplementary Table 6), which were analyzed as we previously described [56].

### HPV detection and identification of integration sites using VirusSeq

For HPV detection and integration analysis, we applied the VirusSeq pipeline previously developed in the Department of Bioinformatics and Computational Biology at The University of Texas MD Anderson Cancer Center [58]. The HPV detection analysis began by preparing reference genomes and annotation files. The reference sequences included human genome hg19 and the virus genomes from the Genome Information Broker for viruses (GIBV, http://gib-v.genes.nig.ac.jp/). Next, the paired-end reads of the samples were aligned against hg19 for human sequence subtraction using the MOSAIK algorithm [59, 60]. Unmapped reads were then aligned against virus genomes to detect the viral genomes. The overall count of the mapped reads in each viral genome was generated, and an empirical cutoff of 1000 read counts mapped within a viral genome was used to distinguish HPV-positive from HPV-negative samples.

To detect HPV integration, the reference genome and annotation files were prepared. The reference genome was a hybrid genome that combined the human hg19 and selected virus genomes containing different types of HPV and other viruses. Paired-end reads were aligned against the hybrid genome, hg19Virus, using MOSAIK to generate files containing all of the discordant reads for each chromosome. The read pair was reported as discordant if the paired-end reads were uniquely mapped with one end to a human chromosome and the other to the virus chromosome. Next, the first mate and the second mate of the paired-end reads were separately aligned against the hybrid hg19Virus genome in order to generate the genomic location for each uniquely mapped read, including information on readID, read sequences, and read mapping orientation. This information was then used for annotating the discordant reads generated previously. A curated refFlat file was used to annotate the discordant reads with human and virus genes. Finally, HPV integration sites were identified using VirusSeq and reported if there were at least 4 supporting discordant read pairs.

### TCGA data analysis

TCGA RNA sequencing files were downloaded from the NCI Genomic Data Commons (https://portal.gdc.cancer.gov/). HPV calls were made as described above and demonstrated 100% concordance with previously published calls [18]. Mutation data were downloaded from the FireBrowse website (http://firebrowse.org/) [61, 62].

### Human and HPV gene expression estimation

To compare global gene expression patterns in HNSCC and CESC cell lines, we performed unsupervised clustering in an unbiased manner using Pearson correlation metrics with Ward’s linkage of the 5000 most variable genes, measured by median absolute deviation, in the 74 cell lines. RNAseq reads were first mapped to human genome hg19 using Tophat/2.0.13 [63]. The read count for human genes was generated using HTSeq [64]. The unmapped reads were then mapped to a combined reference genome of all HPV subtypes. The HPV reference genomes were downloaded from Papillomavirus Episteme database (https://pave.niaid.nih.gov/). The read counts for HPV genes were counted using a Python script developed in house. The reads per kilonucleotide per million-read (rpkm) values were calculated by dividing the read count by the total number of reads in millions and the gene length in kilonucleotides.

## SUPPLEMENTARY MATERIALS FIGURES AND TABLES










