# Supplementary material for: Genomic characterization of human papillomavirus-positive and -negative human squamous cell cancer cell lines
Source: Oncotarget. 2017 Sep 21;8(49):86369–83. doi: 10.18632/oncotarget.21174 (PMC5689691; doi:10.18632/oncotarget.21174)
Supplement: Supplementary file 1 [file oncotarget-08-86369-s001.pdf]

Genomic characterization of human papillomavirus-positive and -negative human squamous cell cancer cell lines

SUPPLEMENTARY MATERIALS

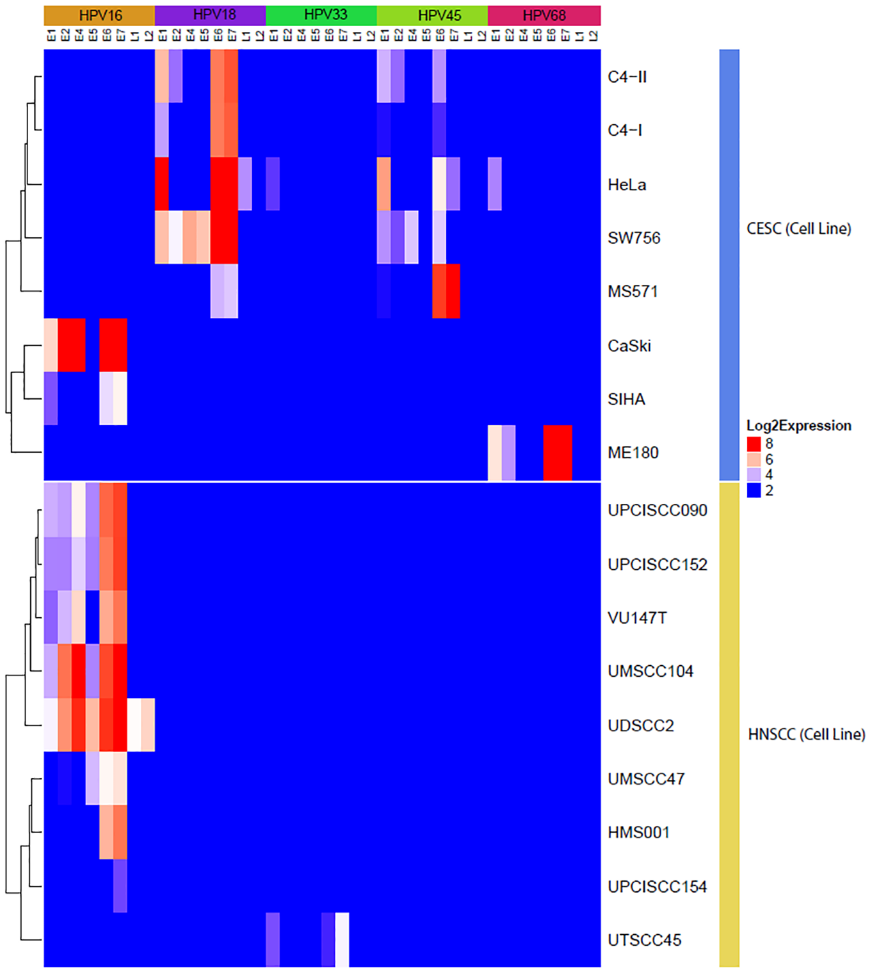

Supplementary Figure 1: HPV viral gene expression in HNSCC and CESC cell lines. Five HPV types are shown: HPV16, HPV18, HPV33, HPV45, and HPV68. Expression levels are represented by the RPKM values (reads per kilonucleotide per million reads).

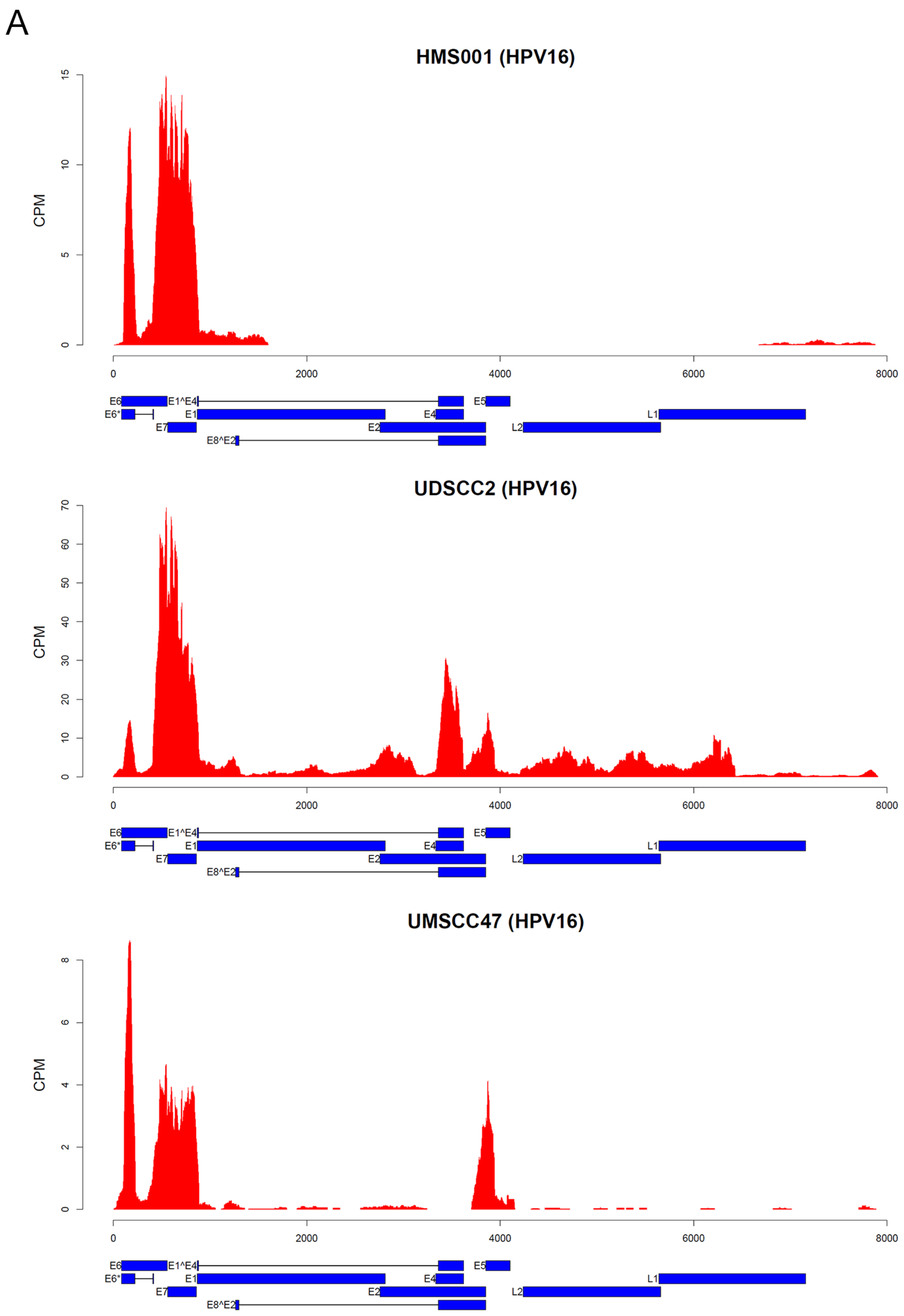
A
HMS001 (HPV16)
CPM
0
5
10
15
0
2000
4000
6000
8000
E6
E1^E4
E5
E6*
E1
E4
L1
E7
E2
L2
E8^E2
UDSCC2 (HPV16)
CPM
0
10
20
30
40
50
60
70
UMSCC47 (HPV16)
CPM
0
2
4
6
8

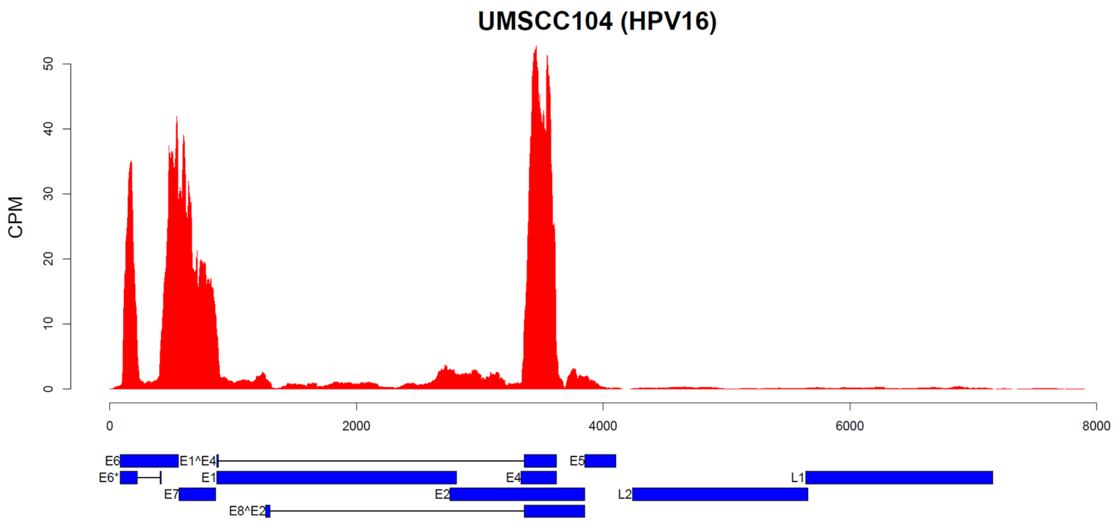
UMSCC104 (HPV16)
CPM
0
10
20
30
40
50
0
2000
4000
6000
8000
E6
E1^E4
E5
E6*
E1
E4
L1
E7
E2
L2
E8^E2

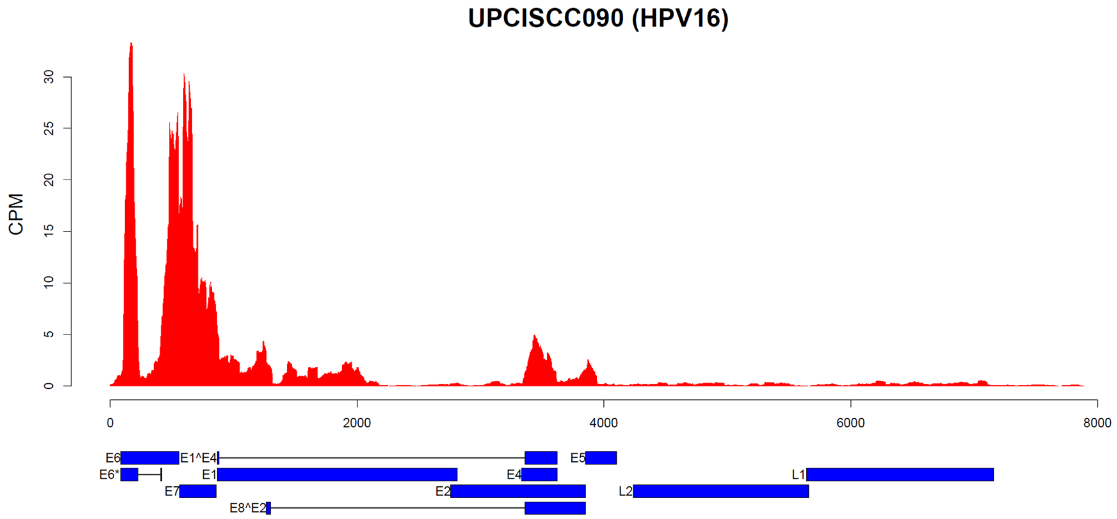
UPCISCC090 (HPV16)
CPM
0
5
10
15
20
25
30
0
2000
4000
6000
8000
E6
E1^E4
E5
E6*
E1
E4
L1
E7
E2
L2
E8^E2

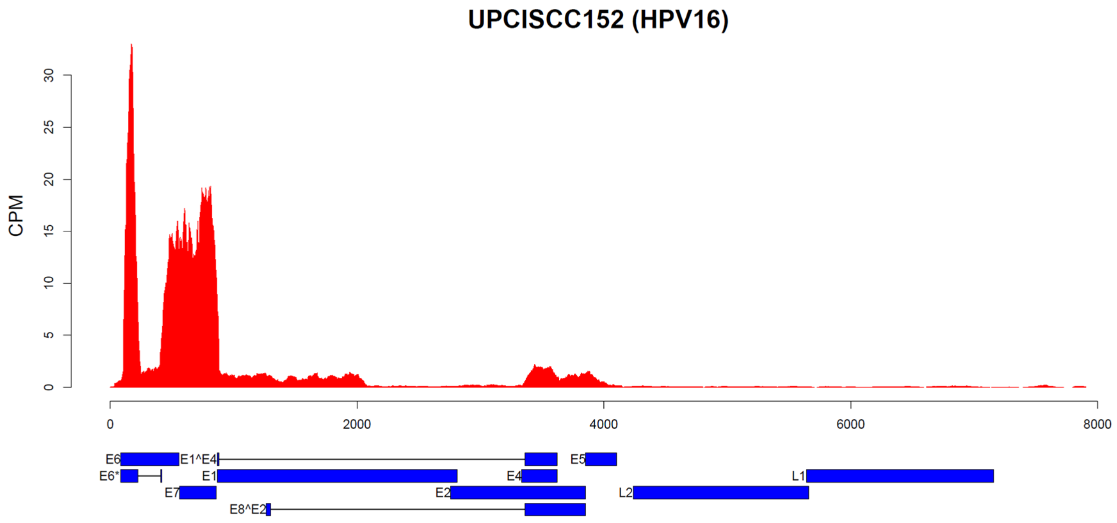
UPCISCC152 (HPV16)
CPM
0
5
10
15
20
25
30
0
2000
4000
6000
8000
E6
E1^E4
E5
E6*
E1
E4
L1
E7
E2
L2
E8^E2

UPCISCC154 (HPV16)

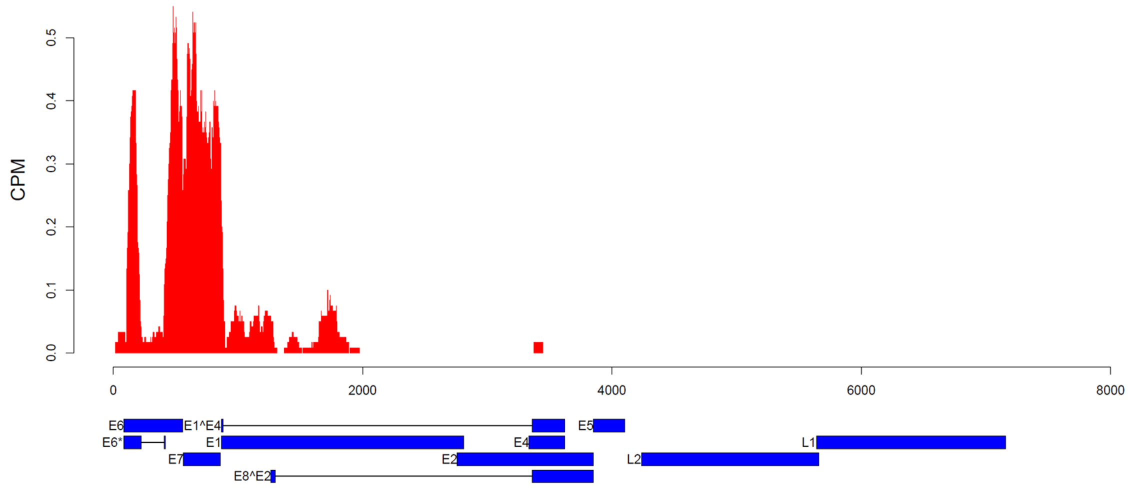

UTSCC45 (HPV33)

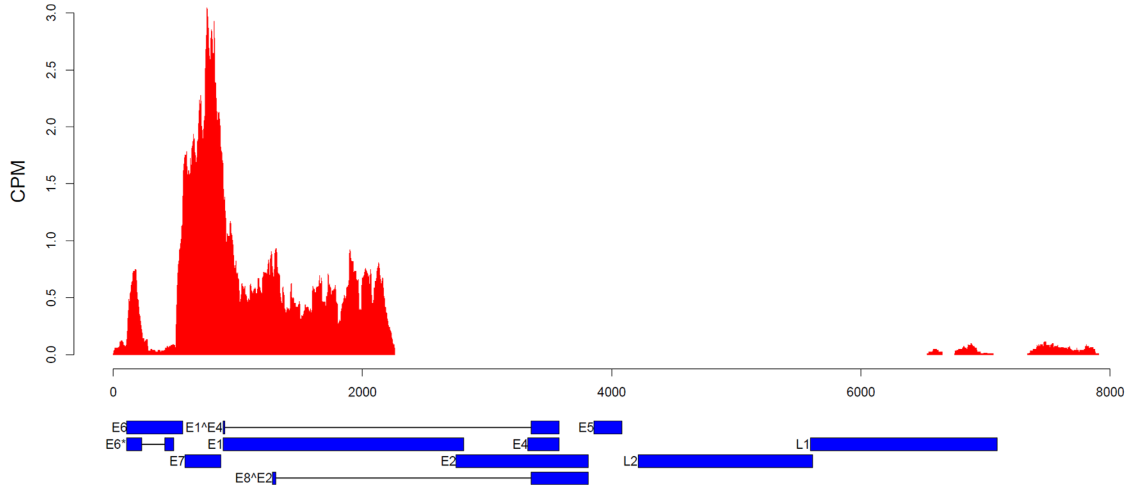

VU147T (HPV16)

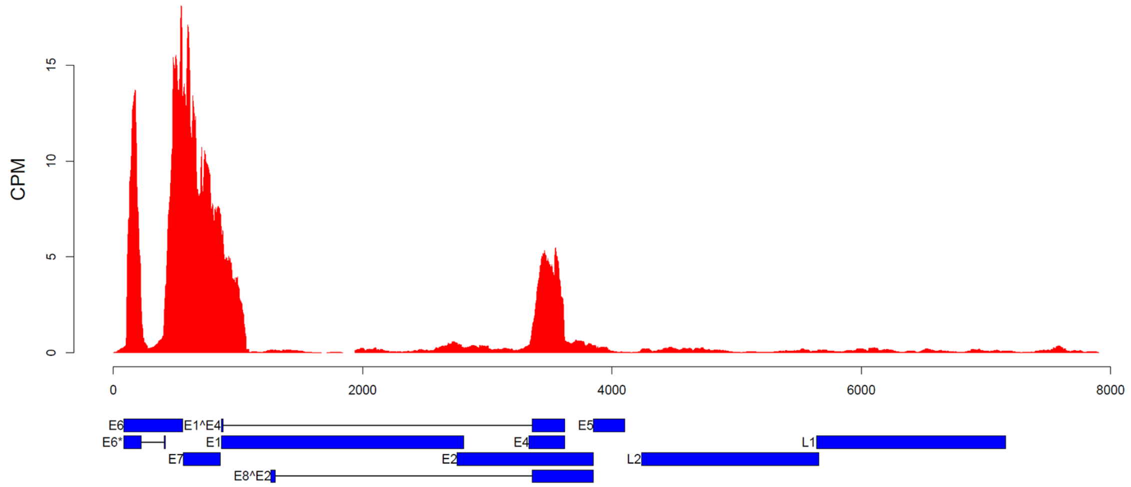

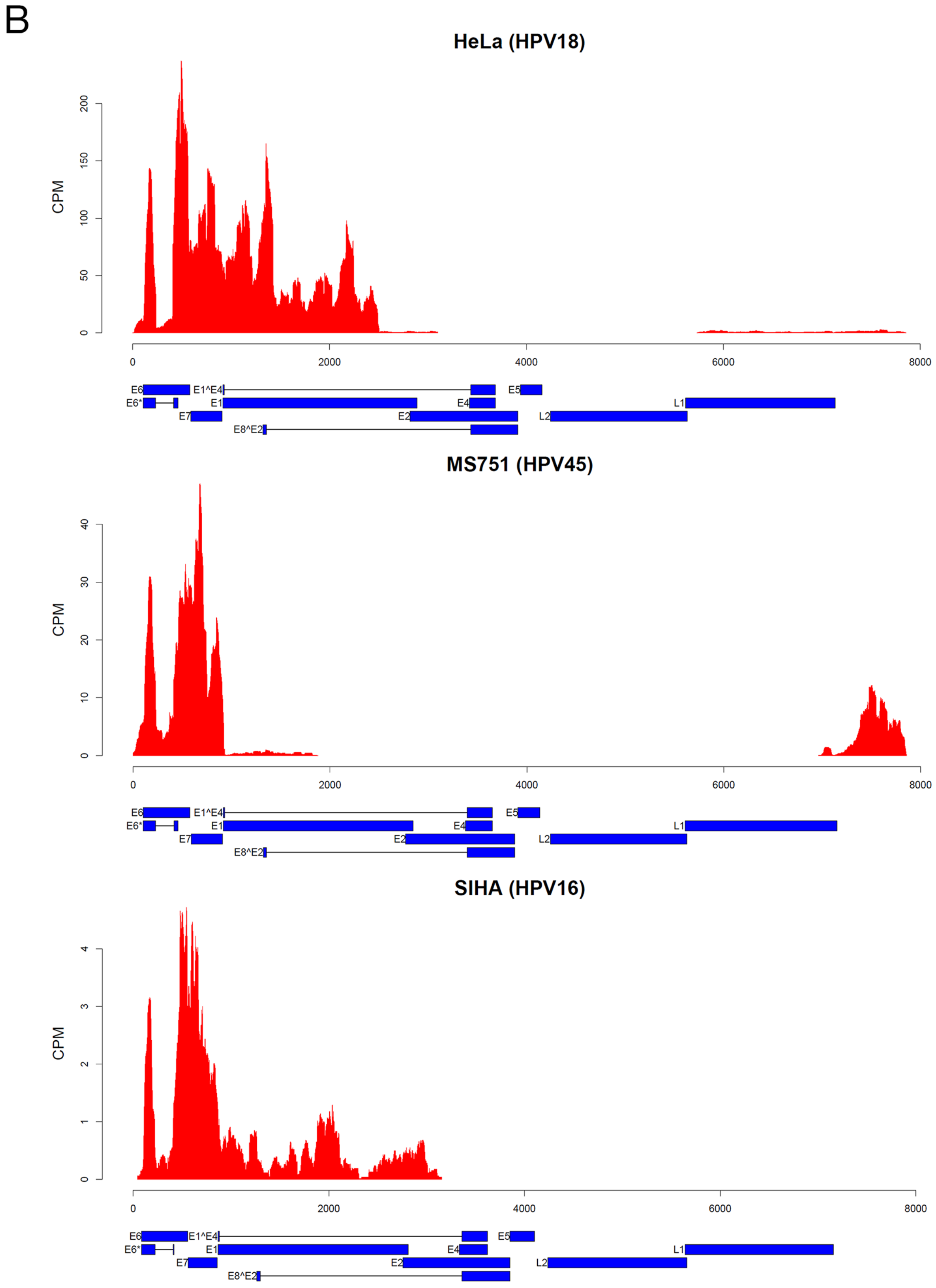
B
HeLa (HPV18)
CPM
0
50
100
150
200
0
2000
4000
6000
8000
E6
E1^E4
E5
E6*
E1
E4
L1
E7
E2
L2
E8^E2
MS751 (HPV45)
CPM
0
10
20
30
40
0
2000
4000
6000
8000
E6
E1^E4
E5
E6*
E1
E4
L1
E7
E2
L2
E8^E2
SIHA (HPV16)
CPM
0
1
2
3
4
0
2000
4000
6000
8000
E6
E1^E4
E5
E6*
E1
E4
L1
E7
E2
L2
E8^E2

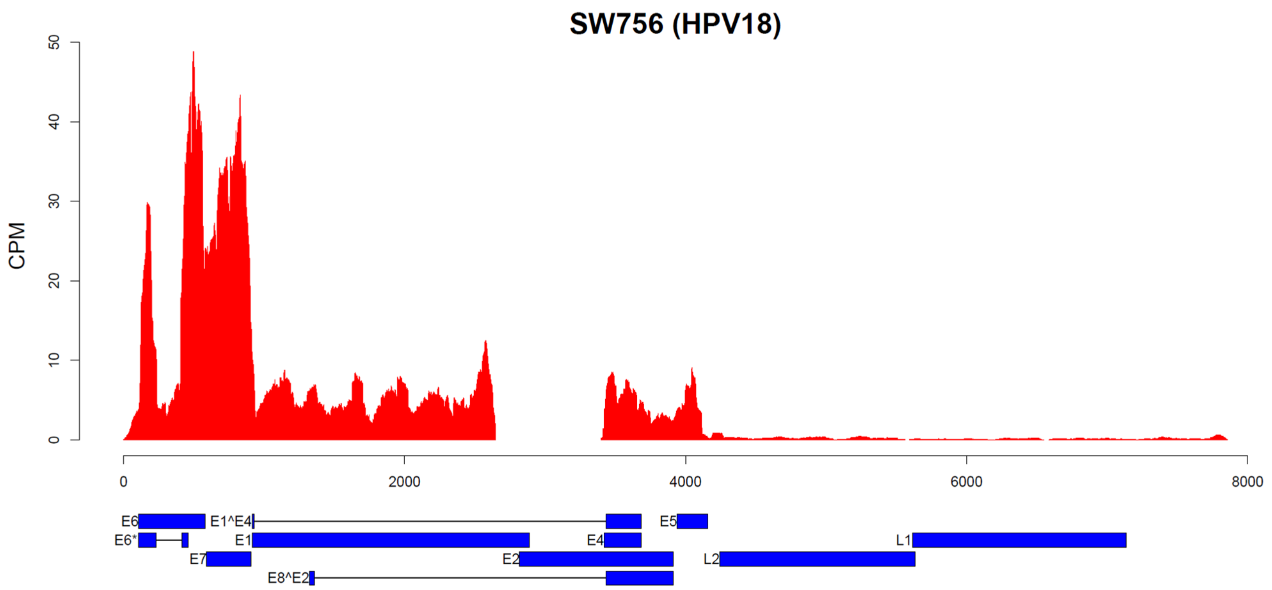
SW756 (HPV18)
CPM
0
10
20
30
40
50
0
2000
4000
6000
8000
E6
E1^E4
E5
E6*
E1
E4
L1
E7
E2
L2
E8^E2

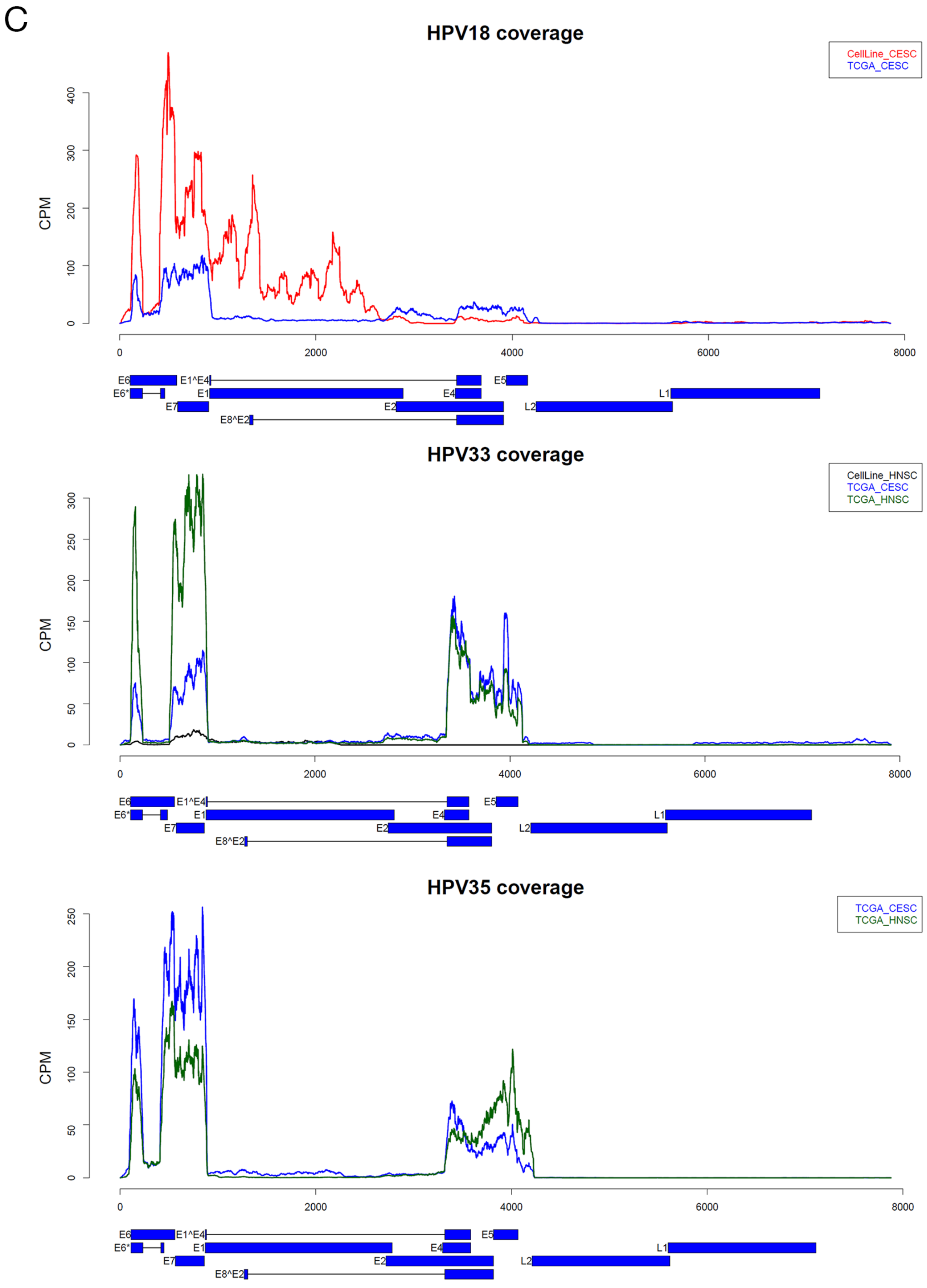

Supplementary Figure 2: HPV viral gene expression in HNSCC and CESC cell lines. Gene expression across the viral genome for each individual HNSCC **(A)** and CESC **(B)** cell line and for the indicated HPV type-positive cell lines and TGCA samples **(C)** is plotted. Expression levels are represented as counts per million reads (CPM).

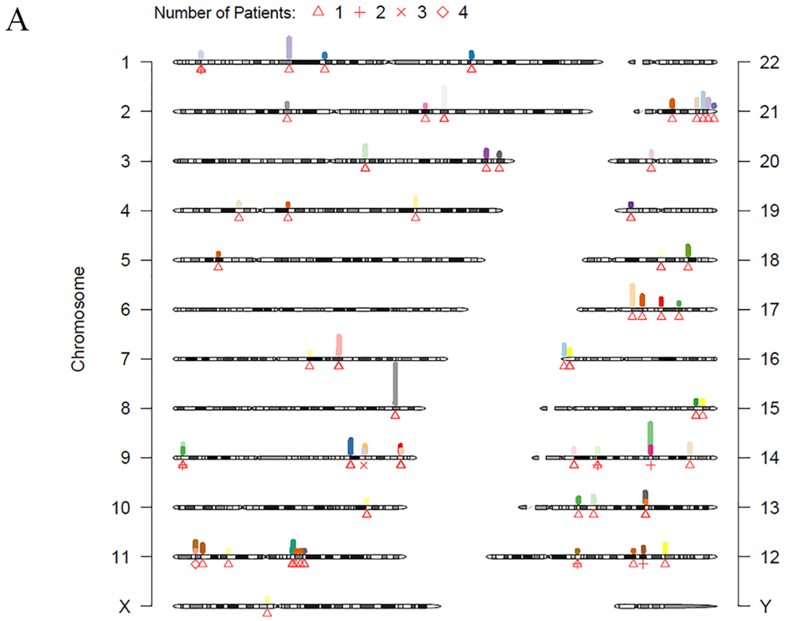

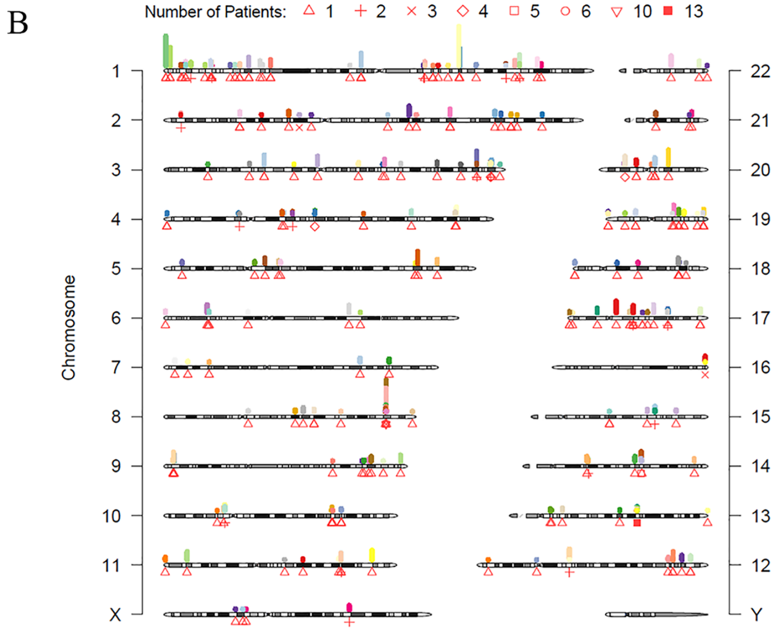

Supplementary Figure 3: HPV integration sites in TCGA tumors. Viral integration sites for 51 HPV-positive HNSCC **(A)** and 194 CESC tumors **(B)** in TCGA are mapped across chromosomes. The symbols below the chromosomes indicate the number of patients with HPV integration in that gene. Above the chromosomes, each color refers to a unique TCGA sample with HPV integration. Because there are many tumors and not that many distinct colors available, some similar colors have to be used. Each bar represents a gene that has HPV integration. Bar length is proportional to the number of integration events/sites for a gene. Note that some bars overlap.

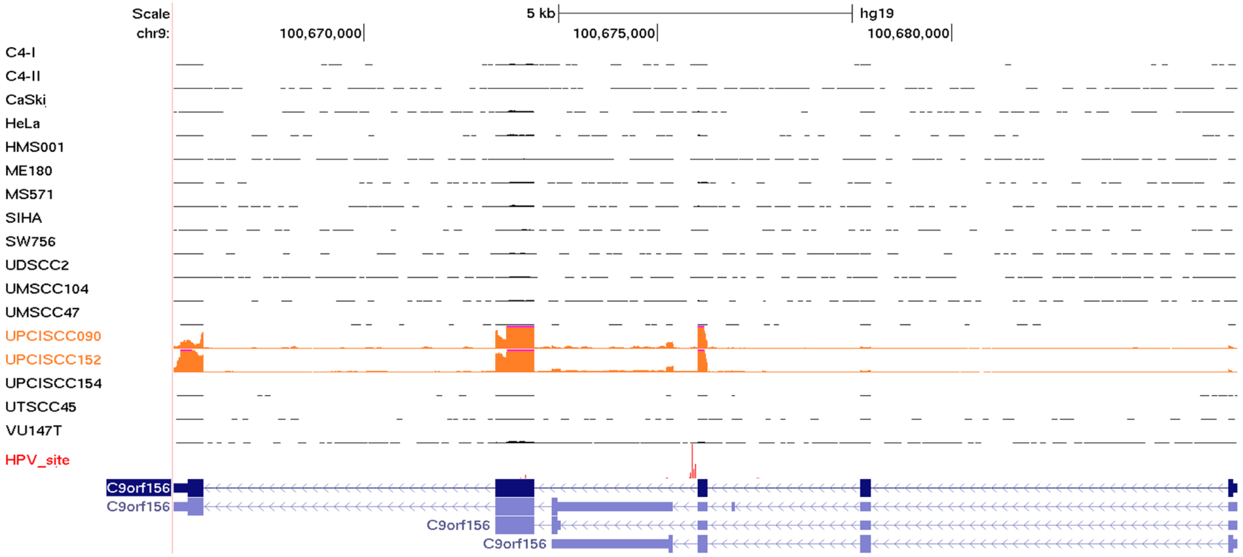
Scale
chr9:
5 kb
hg19
100,670,000
100,675,000
100,680,000
C4-I
C4-II
CaSki
HeLa
HMS001
ME180
MS571
SIHA
SW756
UDSCC2
UMSCC104
UMSCC47
UPCISCC090
UPCISCC152
UPCISCC154
UTSCC45
VU147T
HPV_site
C9orf156
C9orf156
C9orf156
C9orf156

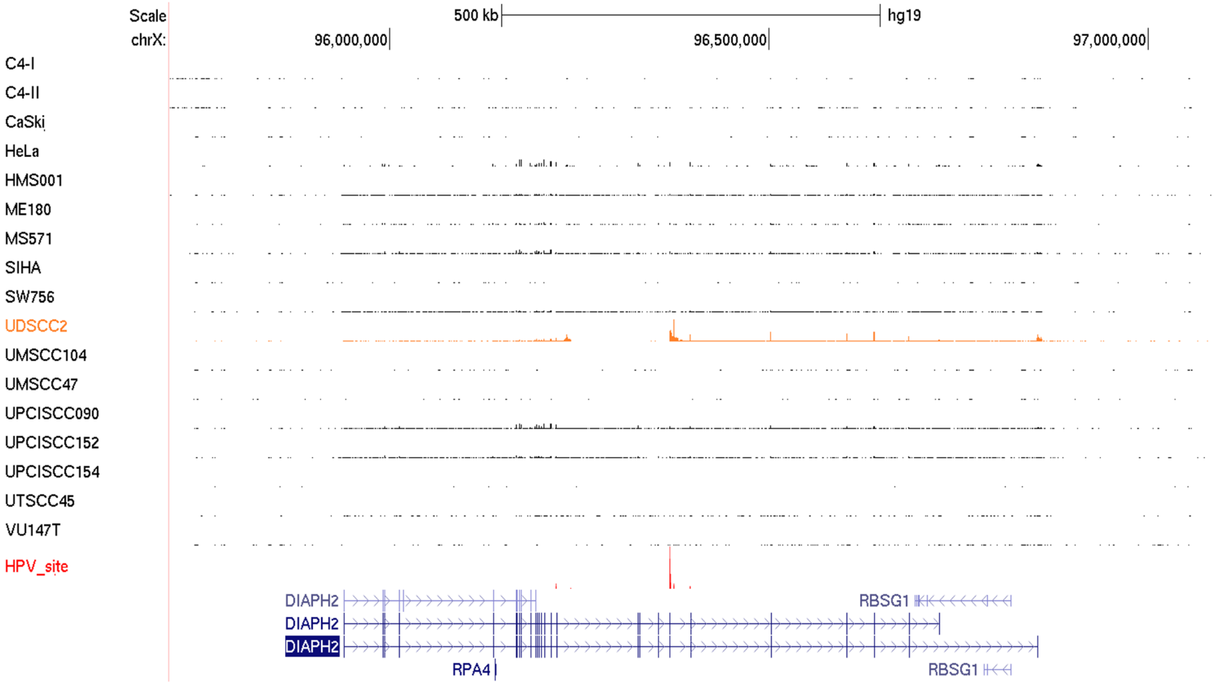
Scale
chrX:
500 kb
hg19
96,000,000
96,500,000
97,000,000
C4-I
C4-II
CaSki
HeLa
HMS001
ME180
MS571
SIHA
SW756
UDSCC2
UMSCC104
UMSCC47
UPCISCC090
UPCISCC152
UPCISCC154
UTSCC45
VU147T
HPV_site
DIAPH2
DIAPH2
DIAPH2
RPA4
RBSG1
RBSG1

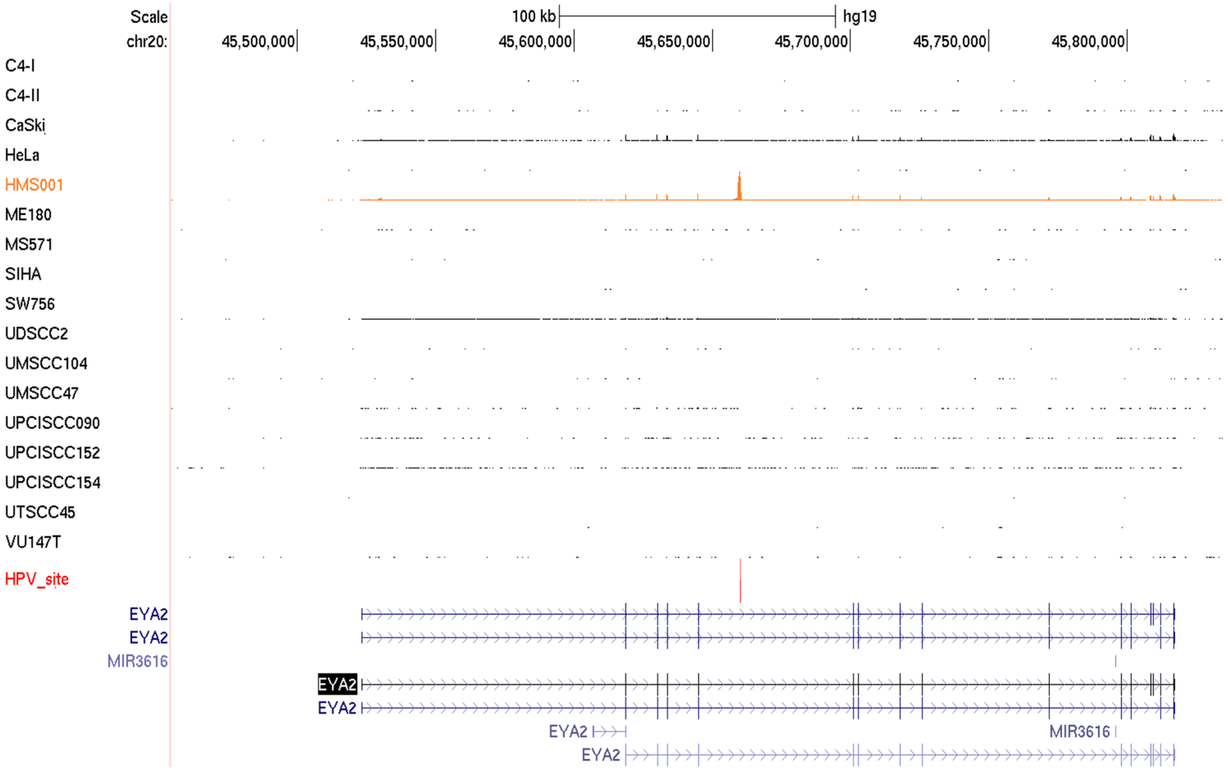
Scale
100 kb
hg19
chr20:
45,500,000
45,550,000
45,600,000
45,650,000
45,700,000
45,750,000
45,800,000
C4-I
C4-II
CaSki
HeLa
HMS001
ME180
MS571
SIHA
SW756
UDSCC2
UMSCC104
UMSCC47
UPCISCC090
UPCISCC152
UPCISCC154
UTSCC45
VU147T
HPV_site
EYA2
EYA2
MIR3616
EYA2
EYA2
EYA2
MIR3616
EYA2

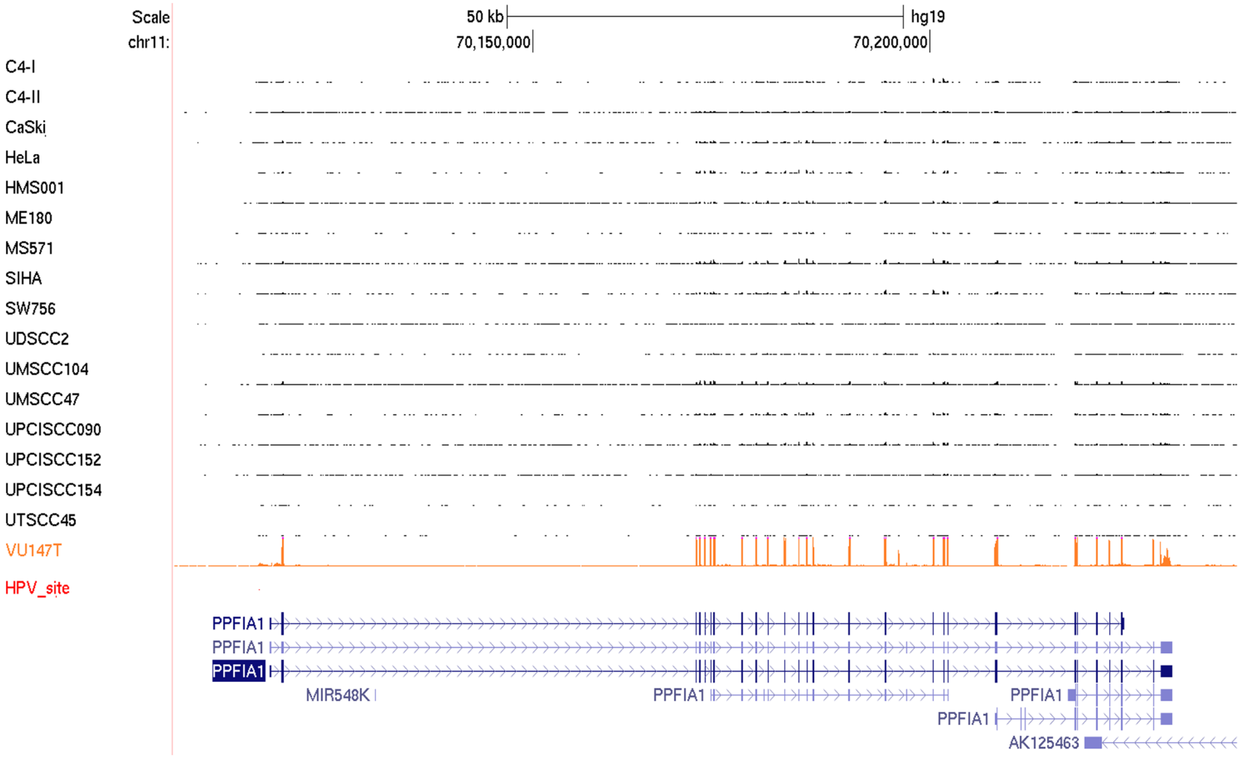
Scale
50 kb
hg19
chr11:
70,150,000
70,200,000
C4-I
C4-II
CaSki
HeLa
HMS001
ME180
MS571
SIHA
SW756
UDSCC2
UMSCC104
UMSCC47
UPCISCC090
UPCISCC152
UPCISCC154
UTSCC45
VU147T
HPV_site
PPFIA1
PPFIA1
PPFIA1
MIR548K
PPFIA1
PPFIA1
PPFIA1
AK125463

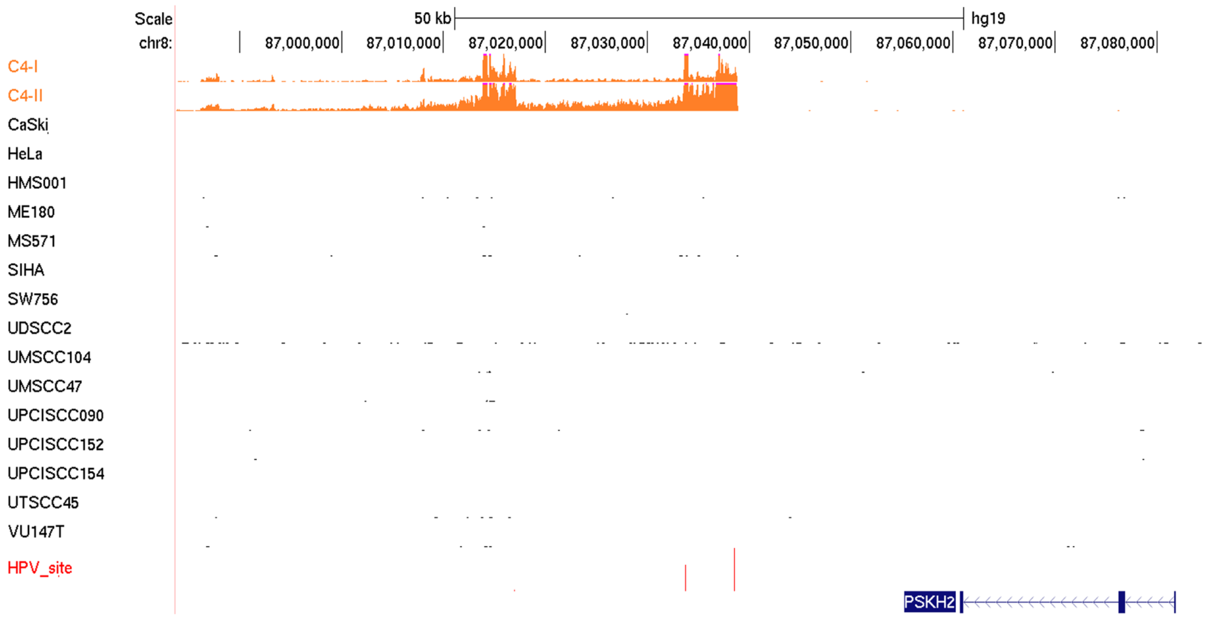
Scale
50 kb
hg19
chr8:
87,000,000
87,010,000
87,020,000
87,030,000
87,040,000
87,050,000
87,060,000
87,070,000
87,080,000
C4-I
C4-II
CaSki
HeLa
HMS001
ME180
MS571
SIHA
SW756
UDSCC2
UMSCC104
UMSCC47
UPCISCC090
UPCISCC152
UPCISCC154
UTSCC45
VU147T
HPV_site
PSKH2

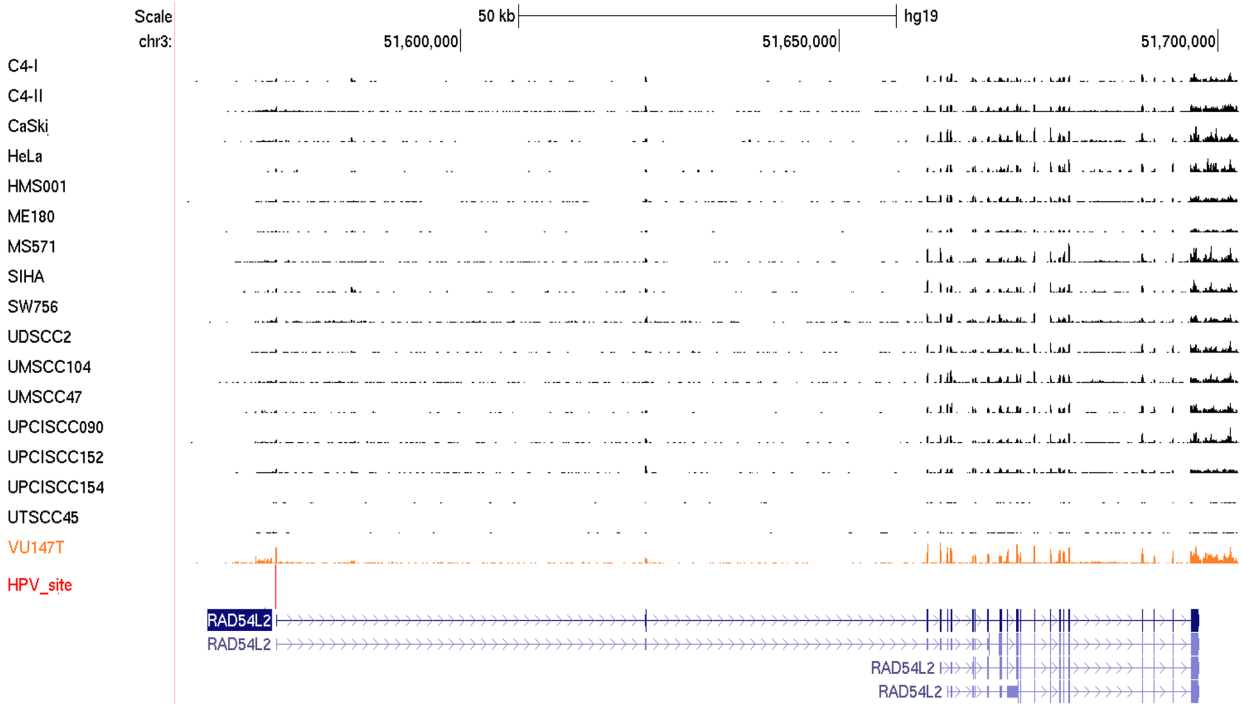
Scale
50 kb
hg19
chr3:
51,600,000
51,650,000
51,700,000
C4-I
C4-II
CaSki
HeLa
HMS001
ME180
MS571
SIHA
SW756
UDSCC2
UMSCC104
UMSCC47
UPCISCC090
UPCISCC152
UPCISCC154
UTSCC45
VU147T
HPV_site
RAD54L2
RAD54L2
RAD54L2
RAD54L2

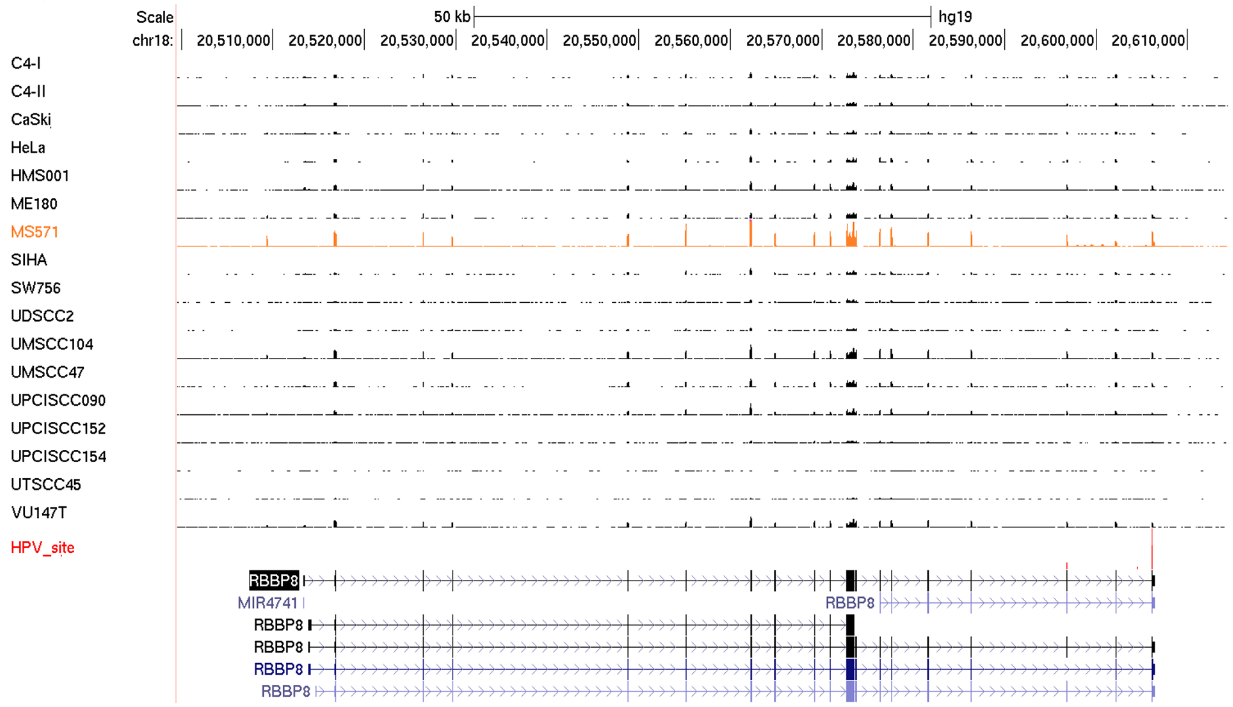
Scale
50 kb
hg19
chr18:
20,510,000
20,520,000
20,530,000
20,540,000
20,550,000
20,560,000
20,570,000
20,580,000
20,590,000
20,600,000
20,610,000
C4-I
C4-II
CaSki
HeLa
HMS001
ME180
MS571
SIHA
SW756
UDSCC2
UMSCC104
UMSCC47
UPCISCC090
UPCISCC152
UPCISCC154
UTSCC45
VU147T
HPV_site
RBBP8
MIR4741
RBBP8
RBBP8
RBBP8
RBBP8
RBBP8

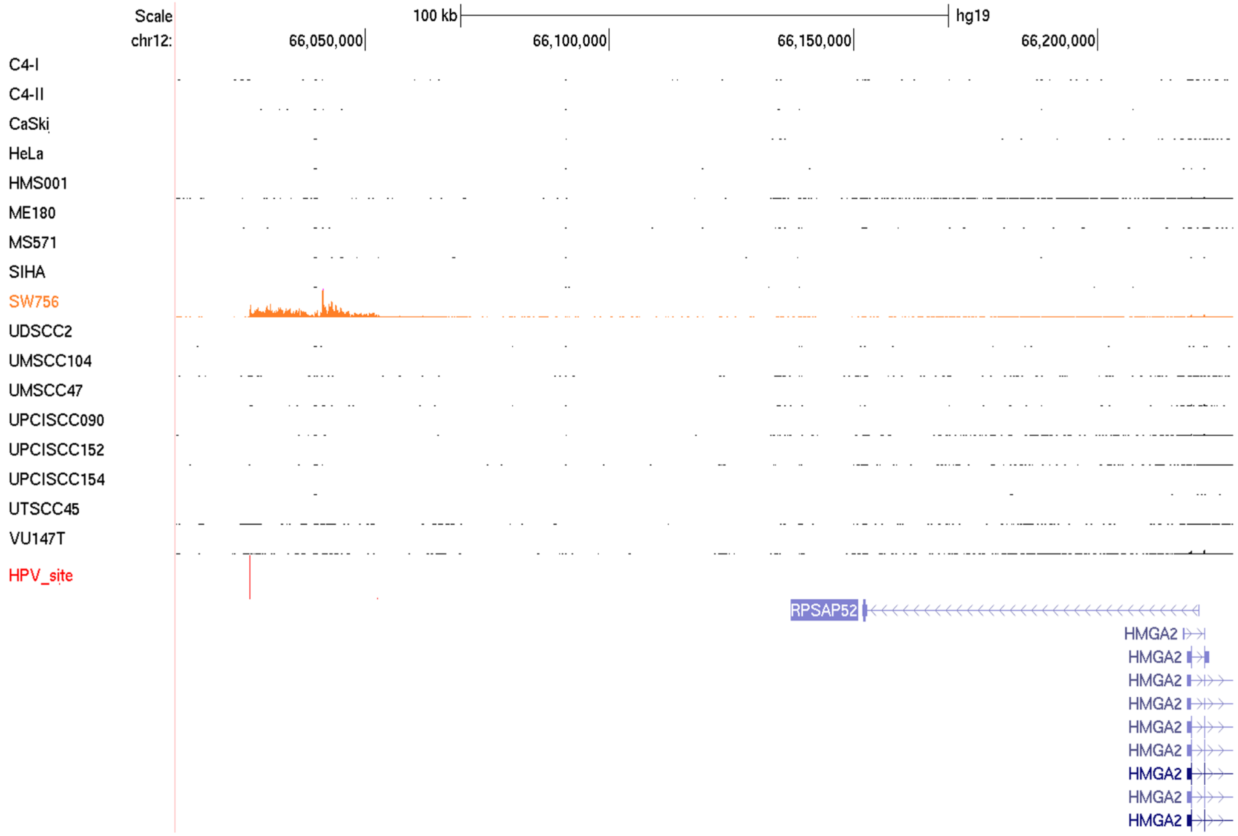
Scale
100 kb
hg19
chr12:
66,050,000
66,100,000
66,150,000
66,200,000
C4-I
C4-II
CaSki
HeLa
HMS001
ME180
MS571
SIHA
SW756
UDSCC2
UMSCC104
UMSCC47
UPCISCC090
UPCISCC152
UPCISCC154
UTSCC45
VU147T
HPV_site
RPSAP52
HMGA2
HMGA2
HMGA2
HMGA2
HMGA2
HMGA2
HMGA2
HMGA2
HMGA2

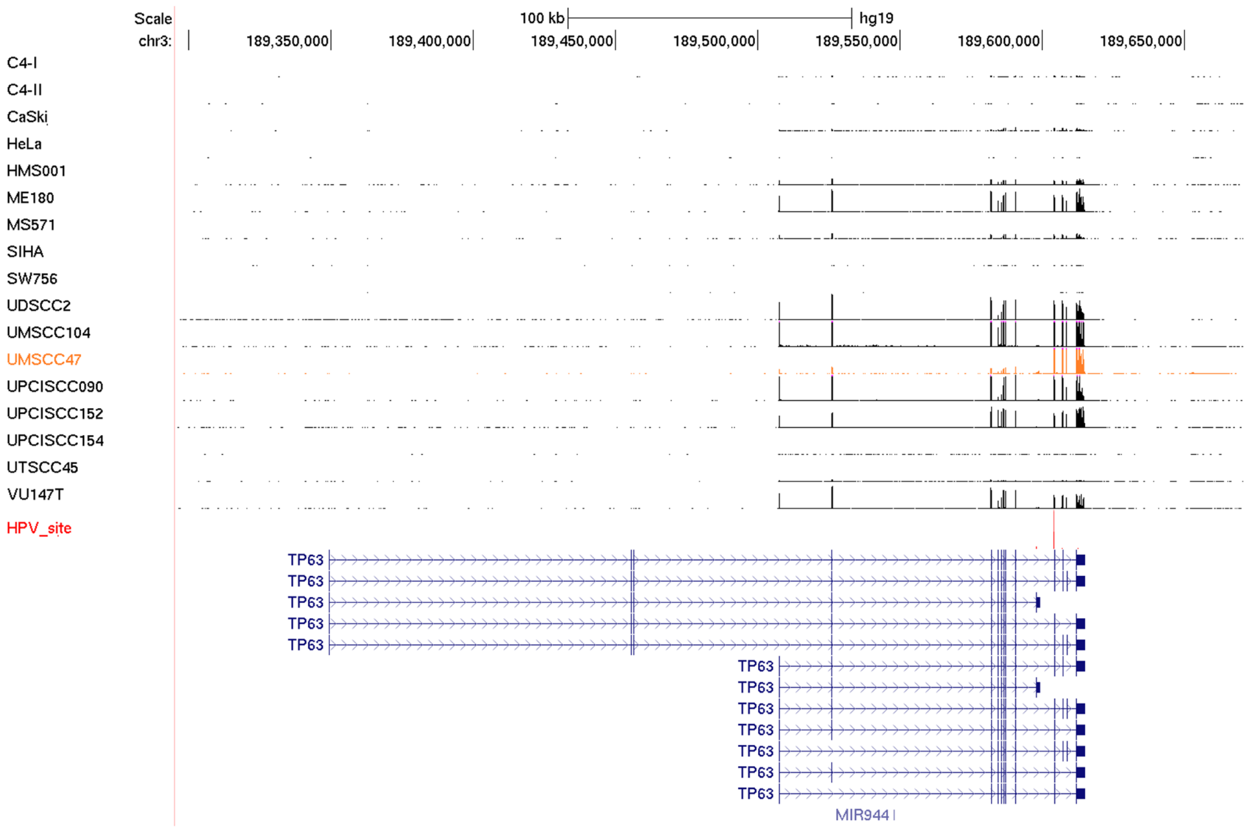
Scale
100 kb
hg19
chr3:
189,350,000
189,400,000
189,450,000
189,500,000
189,550,000
189,600,000
189,650,000
C4-I
C4-II
CaSki
HeLa
HMS001
ME180
MS571
SIHA
SW756
UDSCC2
UMSCC104
UMSCC47
UPCISCC090
UPCISCC152
UPCISCC154
UTSCC45
VU147T
HPV_site
TP63
TP63
TP63
TP63
TP63
TP63
TP63
TP63
TP63
TP63
TP63
TP63
MIR944

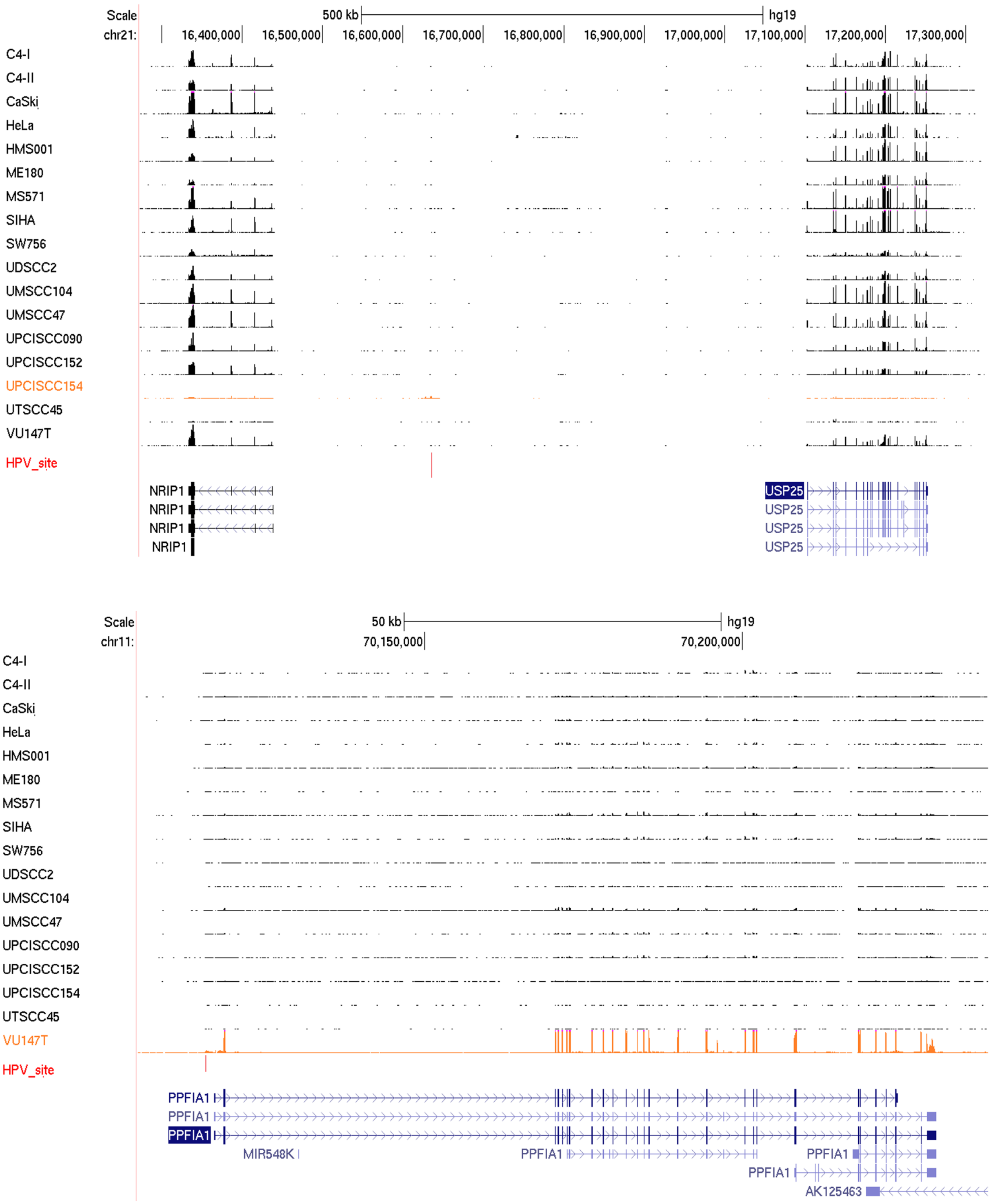

Supplementary Figure 4: HPV integration alters host gene expression. RNASeq reads occupancy profile for the indicated genes in CESC and HNSCC cell lines. Text and data for the cell lines with the HPV integration sites (red vertical lines) detected near these genes appear in orange. Cell lines without integration events near these genes appear in black.

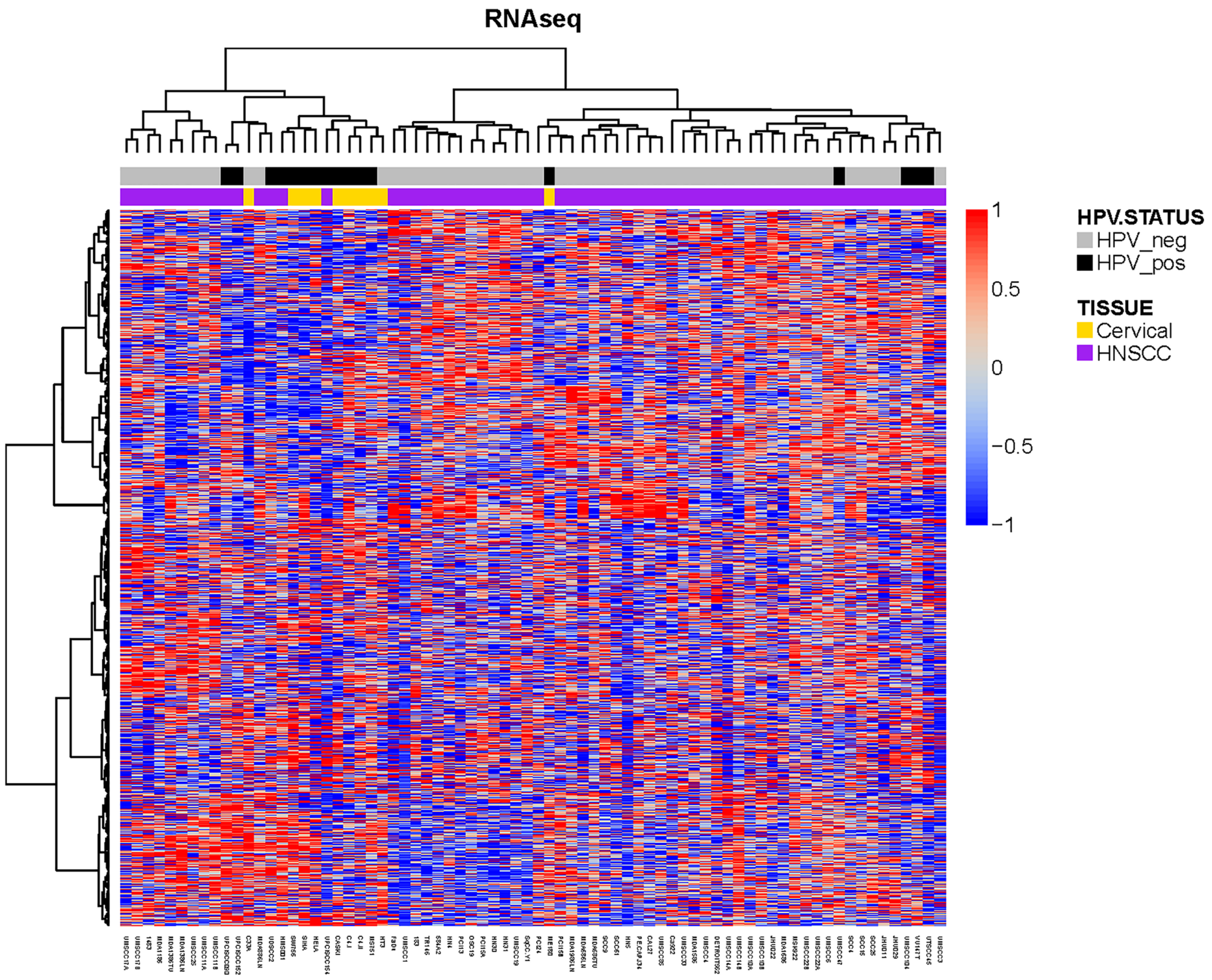

Supplementary Figure 5: Unsupervised clustering of cell lines by gene expression. Unsupervised clustering of all 74 cell lines (columns) using the 5000 most variable genes (rows) with expression measured by RNASeq.

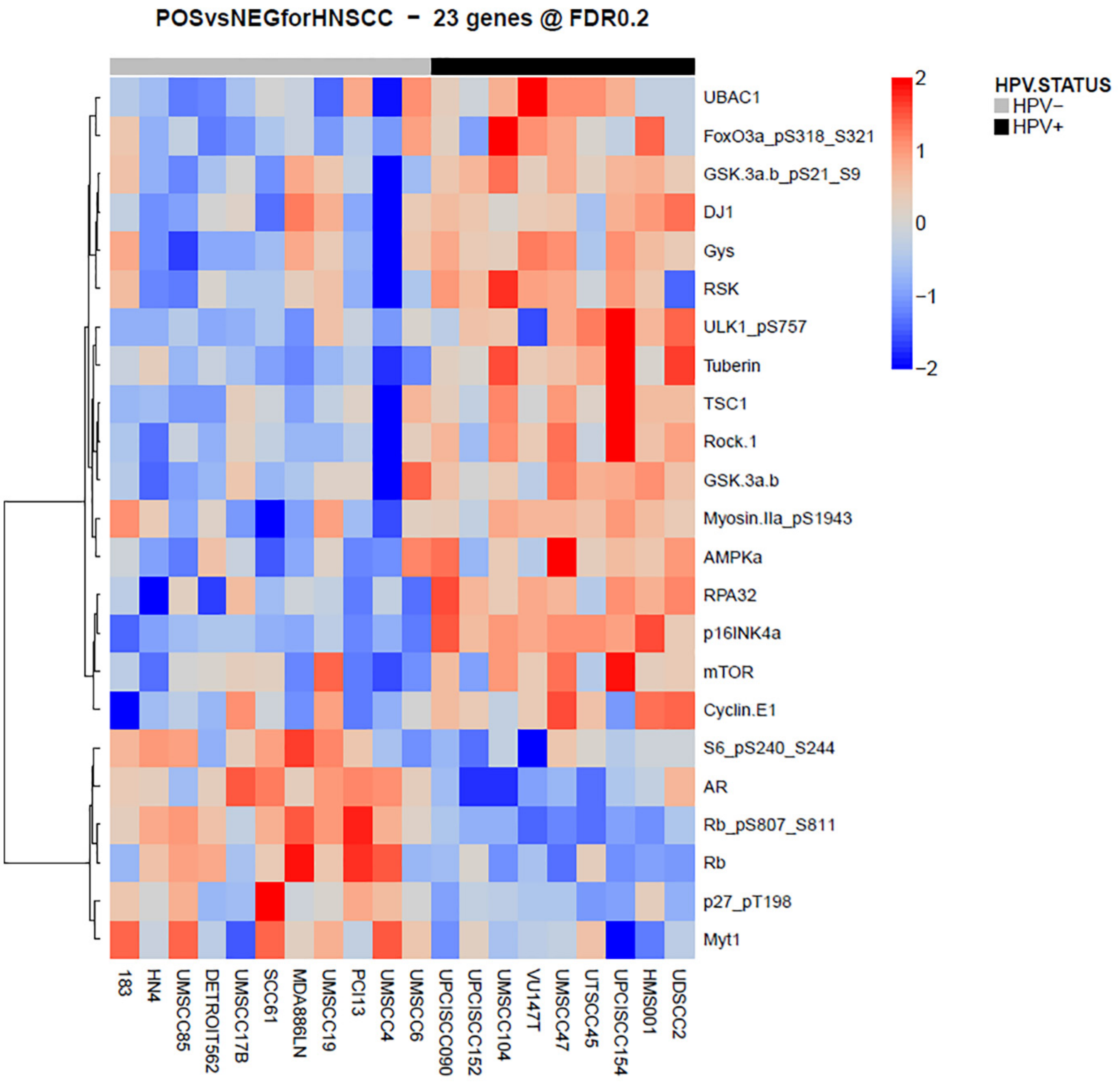

Supplementary Figure 6: Supervised clustering of protein expression by HPV status in HNSCC cell lines. Protein from HPV-positive and HPV-negative HNSCC cell lines was subjected to RPPA proteomic profiling and analyzed by supervised clustering by HPV status. The 23 proteins (rows) that were differentially expressed in HPV-positive and HPV-negative cell lines (columns) are displayed in a heat map.

Supplementary Table 1: The 74 HNSCC and CESC cell lines tested in the study

See Supplementary File 1

Supplementary Table 2: Short tandem repeat analysis for all HPV-positive cell lines

	Loci								
**Cell line**	**AMEL**	**CSF1PO**	**D13S317**	**D16S539**	**D5S818**	**D7S820**	**TH01**	**TPOX**	**vWA**
UMSCC47	X,Y	11,13	8,11	8,13	11,12	11	7,9.3	10,11	18
UPCISCC090	X,Y	11,12	11	12,13	11,12	9,10	7	8	17
UPCISCC152	X,Y	11,12	11	12,13	11,12	9,10	7,9.3	8	17
93-VU-147T	X	11,12	12	9,11	11,12	10,11	7,9	9,11	18
UTSCC45	X	10,12	11,14	9,13	11,12	11,12	7,9.3	11	18,20
UDSCC2	X,Y	11,12	8	11,13	10,11	8,9	8,9	8,10	15,18
UPCISCC154	X,Y	10,12	9,12	13	11,12	9,10	7	8,9	17
HMS001	X,Y	11	8	9,12	11,12	12	6	8	17
UMSCC104	X,Y	10,12	8,9	9,13	12	10	6	9,10	16,17
C4-I	X	12	11,12	11	9,11	10,11	9.3	10,11	14
C4-II	X	12	11,12	11	9,11	10,11	9,9.3	10,11	14
HeLa	X	9,10	12,13.3	9,10	11,12	8,12	7	8,12	16,18
CASKI	X	10	8,12	11,12	13	8,11	7	8	17
SIHA	X	12	11	8,12	9	10	6,9	8,9	14,17
SW756	X	11,12	11	12,13	11,12	10,12	9.3	8	17
MS751	X	11,12	12	11	12	9,11	6	8	16
ME180	X	11	11,13	12,13	12	9,10	8,9.3	8,10	15,17

Supplementary Table 3: Optimal growth conditions, characteristics and doubling times of the HPV-positive HNSCC and CESC cell lines

Cell line	Age	Sex	HPV type	Doubling time (days)	Culture media	Reference
UDSCC2	58	M	16	4.1	DMEM, 10% FBS, NEAA	42
93VU147T	58	M	16	2.2	DMEM, 10% FBS, NEAA	43
UMSCC47	53	M	16	2.2	DMEM, 10% FBS	39
UMSCC104	56	M	16	2.6	DMEM, 10% FBS, NEAA	40
UPCISCC090	46	M	16	2.8	MEM, 10% FBS, NEAA	41
UPCISCC152	47	M	16	2	MEM, 10% FBS, NEAA	44
UPCISCC154	54	M	16	3.8	MEM, 10% FBS, NEAA	44
UTSCC45	76	M	33	1.3	DMEM, 10% FBS, NEAA	44
HMS001	unknown	M	16	5	DMEM/F12, 10% FBS	12
Caski	40	F	16	3.2	RPMI, 10% FBS	24
SiHa	55	F	16	2.6	DMEM, 10% FBS	24
HeLa	31	F	18	1.3	DMEM, 10% FBS	24
MS751	47	F	18	2.4	DMEM, 10% FBS	24
C4-I	41	F	18	2	Waymouth’s MB 752/1, 10% FBS	21
C4-II	41	F	18	2.4	Waymouth’s MB 752/1, 10% FBS	21
SW756	46	F	18	1.6	Leibovitz’s L-15, 10% FBS	45
ME180	66	F	68	1.5	McCoy’s 5a Medium Modified	24
C33	66	F	negative	1.36	Eagles MEM, 10% FBS	21
HT3	58	F	negative	2.48	McCoy’s 5a Medium Modified, 10% FBS	21

Supplementary Table 4: Integration in HPV-positive cell lines

See Supplementary File 2

Supplementary Table 5: Genes with recurrent integration in HPV positive cell lines and TCGA tumor samples

See Supplementary File 3

Supplementary Table 6: Antibodies (Ab) used for reverse phase protein array (RPPA) analysis

See Supplementary File 4

Supplementary Table 7: Proteins that were differentially expressed by HPV status at a FDR of 0.2

Protein	p value	Adjusted p value
UBAC1	0.0172	0.2366
pFoxO3A(S318/321)	0.0163	0.2366
pGSK.3a.b(S21/S9)	0.0038	0.1439
DJ1	0.0168	0.2366
Gys	0.0196	0.2577
RSK	0.0089	0.2154
pULK1(S757)	0.0047	0.1577
Tuberin	0.0001	0.0130
TSC1	0.0013	0.0581
Rock1	0.0011	0.0577
GSK.3a.b	0.0065	0.1967
pMyosin11A(S1943)	0.0092	0.2154
AMPKa	0.0143	0.2366
RPA32	0.0002	0.0130
p16INK4a	0.0000	0.0000
mTOR	0.0137	0.2366
CyclinE1	0.0150	0.2366
pS6(S240/244)	0.0159	0.2366
AR	0.0086	0.2154
pRb(S807/811)	0.0000	0.0000
Rb	0.0007	0.0420
p27(T198)	0.0166	0.2366
Myt1	0.0153	0.2366

Supplementary Table 8: The sequence of PCR primers for HPV E6 and E7

Primer Name	Primer Sequence (5' to 3')
HPV - 16 - E7 - Sense	TTGTTGCAAGTGTGACTCTACGC
HPV - 16 - E7 - Anti-Sense	CCTAGTGTGCCCATTAACAGGTC
HPV - 16 - E6 - Sense	GCAATGTTTCAGGACCCACA
HPV - 16 - E6 - Anti-Sense	CGCAGTAACTGTTGCTTGCAGT
HPV - 18 - E7 - Sense	ATGAAATTCCGGTTGACCTTC
HPV - 18 - E7 - Anti-Sense	GTCGGGCTGGTAAATGTTGAT
HPV - 18 - E6 - Sense	TCACAACATAGCTGGGCACTA
HPV - 18 - E6 - Anti-Sense	CTTGTGTTTCTCTGCGTCGTT
HPV - 16 - Sense	GTGGACCGGTCGATGTATGTCT
HPV - 16 - Anti-Sense	TCCGGTTCTGCTTGTCCAGC
HPV - 18 - Sense	AGTGCCATTCGTGCTGCAAC
HPV - 18 - Anti - Sense	ATGTTGCCTTAGGTCCATGCAT
HPV - 33 - Sense	AATATTTCGGGTCGTTGGGC
HPV - 33 - Anti-Sense	AACGTTGGCTTGTGTCCTCTCA
HPV - 45 - Sense	GGACAGTACCGAGGGCAGTGTA
HPV - 45 - Anti-Sense	CCGGGGTCCATGCATACTTAT
GAPDH-Sense	CACCATTGGCAATGAGCGGTTC
GAPDH-Anti-sense	AGGTCTTTGCGGATGTCCACGT
