# Supplementary material for: Genomic characterization of human papillomavirus-positive and -negative human squamous cell cancer cell lines
Source: Oncotarget. 2017 Sep 21;8(49):86369–83. doi: 10.18632/oncotarget.21174 (PMC5689691; doi:10.18632/oncotarget.21174)
Supplement: Supplementary file 2 [file oncotarget-08-86369-s002.docx]

**Supplementary Table 1.** The 74 HNSCC and CESC cell lines tested in the study.

| **Cell line** | **Tumor source** | **HPV status** |
| --- | --- | --- |
| HN4 | REC (L) | Negative |
| HN5 | REC (OC) | Negative |
| HN30 | P | Negative |
| HN31 | LN (HN30) | Negative |
| UM-SCC-1 | REC (OC) | Negative |
| UM-SCC-3 | LN (nose) | Negative |
| UM-SCC-4 | OP | Negative |
| UM-SCC-6 | OP | Negative |
| UM-SCC-10A | L | Negative |
| UM-SCC-10B | LN (UM-SCC-10A) | Negative |
| UM-SCC-14A | REC (OC) | Negative |
| UM-SCC-14B | REC (UM-SCC-14A) | Negative |
| UM-SCC-17A | L | Negative |
| UM-SCC-17B | EXT (UM-SCC-17A) | Negative |
| UM-SCC-19 | OP | Negative |
| UM-SCC-22A | HP | Negative |
| UM-SCC-22B | LN (UM-SCC-22A) | Negative |
| UM-SCC-25 | LN (L) | Negative |
| UM-SCC-33 | LN (maxillary sinus) | Negative |
| UM-SCC-85 | REC (nose) | Negative |
| JHU011 | REC (L) | Negative |
| JHU022 | LN (L) | Negative |
| JHU029 | L | Negative |
| MDA686TU | OP | Negative |
| MDA686LN | LN (MDA686TU) | Negative |
| MDA886LN | LN (L) | Negative |
| MDA1186 | L | Negative |
| MDA1386TU | HP | Negative |
| MDA1386LN | LN (MDA1386TU) | Negative |
| MDA1586 | L | Negative |
| MDA1686 | Cheek | Negative |
| PCI15A | HP | Negative |
| PCI15B | LN (PCI-15A) | Negative |
| PCI24 | OC | Negative |
| SCC-4 | OC | Negative |
| SCC-9 | OC | Negative |
| SCC-15 | OC | Negative |
| SCC-25 | OC | Negative |
| SCC-61 | OC | Negative |
| FaDu | HP | Negative |
| OSC19 | LN (OC) | Negative |
| TR146 | REC (OC) | Negative |
| SqCC/Y1 | OC | Negative |
| CAL-27 | OC | Negative |
| MSK-922 | REC (L) | Negative |
| PE/CA-PJ34 | OC | Negative |
| Detroit562 | Pleural effusion (P) | Negative |
| 183 | OP | Negative |
| 1483 | OC | Negative |
| 584A2 | L | Negative |
| MDA1986LN | OC | Negative |
| PCI13 | OC | Negative |
| Ca922 | Gingival SCC | Negative |
| UM-SCC-11A | L | Negative |
| UM-SCC-11B | L | Negative |
| 93-VU-147T | OC | HPV16 |
| UD-SCC2 | HP | HPV16 |
| UPCI: SCC-090 | OC | HPV16 |
| UPCI: SCC-152 | HP | HPV16 |
| UPCI: SCC-154 | OC | HPV16 |
| UM SCC47 | OC | HPV16 |
| UMSCC104 | REC (OC) | HPV16 |
| UT-SCC-45 | OC | HPV33 |
| HMS-001 | OC | HPV16 |
| C33 | Cervix | Negative |
| HT3 | Cervix | Negative |
| C4I | Cervix | HPV18 |
| C4II | Cervix | HPV18 |
| CaSki | Cervix | HPV16^*^ |
| HeLa | Cervix | HPV18 |
| ME180 | Cervix | HPV68^*^ |
| MS751 | Cervix | HPV45^*^ |
| SiHa | Cervix | HPV16 |
| SW756 | Cervix | HPV18 |

REC, recurrence; L, larynx; OC, oral cavity; P, pharynx; LN, lymph node; OP, oropharynx; EXT, extension to adjacent tissue; HP, hypopharynx; SCC, squamous cell carcinoma.

***** In the literature the HPV type for ME180 cells is variably reported as HPV18, HPV39, or HPV68; MS751 as HPV18 and HPV45; and CaSki cells as HPV16 and HPV18. See text for details.
