# Supplementary material for: Genomic characterization of human papillomavirus-positive and -negative human squamous cell cancer cell lines
Source: Oncotarget. 2017 Sep 21;8(49):86369–83. doi: 10.18632/oncotarget.21174 (PMC5689691; doi:10.18632/oncotarget.21174)
Supplement: Supplementary file 3 [file oncotarget-08-86369-s003.docx]

**Supplementary Table 4.** Integration in HPV-positive cell lines

| **Viral Transcript** | **Host Gene** | **Discordant Read Pairs** | **Junction Spanning Reads** | **Gene ChrID** | **Gene Integration Location** | **Sample** | **Cell Type** |
| --- | --- | --- | --- | --- | --- | --- | --- |
| HpV18 E6 | PSKH2 | 4 | 2 | 8 | 87016962 | C4-II | Cervical |
| HpV18 E2 | PSKH2 | 25 | 17 | 8 | 87038582 | C4-II | Cervical |
| HpV18 E7 | PSKH2 | 80 | 11 | 8 | 87033746 | C4-I | Cervical |
| HpV16 E2 | CLIC5 | 53 | 30 | 6 | 45658843 | CaSki | Cervical |
| HpV16 E4 | CLIC5 | 67 | 35 | 6 | 45658983 | CaSki | Cervical |
| HpV18 E6 | POU5F1B | 29 | 7 | 8 | 128231046 | HeLa | Cervical |
| HpV18 E7 | POU5F1B | 212 | 21 | 8 | 128241129 | HeLa | Cervical |
| HpV18 E1 | POU5F1B | 365 | 36 | 8 | 128241258 | HeLa | Cervical |
| HpV18 E2 | POU5F1B | 12 | 8 | 8 | 128233283 | HeLa | Cervical |
| HpV45 E6 | RBBP8 | 104 | 66 | 18 | 20606104 | MS571 | Cervical |
| HpV45 E7 | RBBP8 | 236 | 117 | 18 | 20606101 | MS571 | Cervical |
| HpV45 E1 | RBBP8 | 91 | 86 | 18 | 20606103 | MS571 | Cervical |
| HpV45 E1 | RBBP8 | 17 | 9 | 18 | 20604483 | MS571 | Cervical |
| HpV45 E1 | RBBP8 | 4 | 2 | 18 | 20606102 | MS571 | Cervical |
| HpV45 L1 | RBBP8 | 28 | 20 | 18 | 20596786 | MS571 | Cervical |
| HpV16 E7 | KLF12 | 5 | 2 | 13 | 74051273 | SIHA | Cervical |
| HpV18 E1 | RPSAP52 | 93 | 27 | 12 | 66026434 | SW756 | Cervical |
| HpV18 E4 | RPSAP52 | 27 | 3 | 12 | 66052598 | SW756 | Cervical |
| HpV16 E7 | EYA2 | 166 | 77 | 20 | 45660164 | HMS001 | HNSCC |
| HpV16 E1 | EYA2 | 9 | 4 | 20 | 45660165 | HMS001 | HNSCC |
| HpV16 E6 | DIAPH2 | 10 | 0 | 23 | 96219946 | UDSCC2 | HNSCC |
| HpV16 E6 | DIAPH2 | 36 | 19 | 23 | 96369874 | UDSCC2 | HNSCC |
| HpV16 E6 | DIAPH2 | 4 | 2 | 23 | 96375084 | UDSCC2 | HNSCC |
| HpV16 E7 | DIAPH2 | 14 | 6 | 23 | 96370340 | UDSCC2 | HNSCC |
| HpV16 E2 | DIAPH2 | 31 | 8 | 23 | 96369877 | UDSCC2 | HNSCC |
| HpV16 E4 | DIAPH2 | 4 | 2 | 23 | 96220085 | UDSCC2 | HNSCC |
| HpV16 E4 | DIAPH2 | 9 | 2 | 23 | 96369874 | UDSCC2 | HNSCC |
| HpV16 E4 | DIAPH2 | 4 | 1 | 23 | 96375067 | UDSCC2 | HNSCC |
| HpV16 E4 | DIAPH2 | 5 | 0 | 23 | 96370414 | UDSCC2 | HNSCC |
| HpV16 L2 | DIAPH2 | 10 | 0 | 23 | 96238654 | UDSCC2 | HNSCC |
| HpV16 L1 | DIAPH2 | 5 | 1 | 23 | 96396664 | UDSCC2 | HNSCC |
| HpV16 E2 | SLC47A2 | 4 | 4 | 17 | 19609393 | UMSCC104 | HNSCC |
| HpV16 E2 | SLC47A2 | 105 | 51 | 17 | 19609643 | UMSCC104 | HNSCC |
| HpV16 E4 | SLC47A2 | 143 | 115 | 17 | 19608409 | UMSCC104 | HNSCC |
| HpV16 E4 | SLC47A2 | 20 | 3 | 17 | 19609393 | UMSCC104 | HNSCC |
| HpV16 E6 | TP63 | 189 | 94 | 3 | 189604181 | UMSCC47 | HNSCC |
| HpV16 E7 | TP63 | 20 | 6 | 3 | 189597866 | UMSCC47 | HNSCC |
| HpV16 E7 | TP63 | 104 | 26 | 3 | 189604177 | UMSCC47 | HNSCC |
| HpV16 E7 | TP63 | 6 | 2 | 3 | 189607126 | UMSCC47 | HNSCC |
| HpV16 E1 | TP63 | 5 | 1 | 3 | 189604174 | UMSCC47 | HNSCC |
| HpV16 E2 | TP63 | 5 | 2 | 3 | 189612728 | UMSCC47 | HNSCC |
| HpV16 E5 | TP63 | 4 | 1 | 3 | 189612731 | UMSCC47 | HNSCC |
| HpV16 E6 | C9orf156 | 4 | 1 | 9 | 100675168 | UPCISCC090 | HNSCC |
| HpV16 E6 | C9orf156 | 8 | 0 | 9 | 100675539 | UPCISCC090 | HNSCC |
| HpV16 E6 | C9orf156 | 262 | 47 | 9 | 100675560 | UPCISCC090 | HNSCC |
| HpV16 E7 | C9orf156 | 34 | 28 | 9 | 100672750 | UPCISCC090 | HNSCC |
| HpV16 E7 | C9orf156 | 7 | 1 | 9 | 100675164 | UPCISCC090 | HNSCC |
| HpV16 E7 | C9orf156 | 318 | 115 | 9 | 100675635 | UPCISCC090 | HNSCC |
| HpV16 E1 | C9orf156 | 4 | 0 | 9 | 100676680 | UPCISCC090 | HNSCC |
| HpV16 E4 | FOXE1 | 11 | 8 | 9 | 100615158 | UPCISCC090 | HNSCC |
| HpV16 E6 | C9orf156 | 7 | 0 | 9 | 100675167 | UPCISCC152 | HNSCC |
| HpV16 E6 | C9orf156 | 112 | 78 | 9 | 100675619 | UPCISCC152 | HNSCC |
| HpV16 E7 | C9orf156 | 4 | 0 | 9 | 100672672 | UPCISCC152 | HNSCC |
| HpV16 E7 | C9orf156 | 6 | 4 | 9 | 100675148 | UPCISCC152 | HNSCC |
| HpV16 E7 | C9orf156 | 21 | 8 | 9 | 100675538 | UPCISCC152 | HNSCC |
| HpV16 E7 | C9orf156 | 711 | 289 | 9 | 100675592 | UPCISCC152 | HNSCC |
| HpV16 E1 | C9orf156 | 20 | 0 | 9 | 100676710 | UPCISCC152 | HNSCC |
| HpV16 E4 | FOXE1 | 4 | 1 | 9 | 100615201 | UPCISCC152 | HNSCC |
| HpV16 E7 | USP25 | 7 | 0 | 21 | 16635859 | UPCISCC154 | HNSCC |
| HpV33 E1 | BIN1 | 6 | 1 | 2 | 127500913 | UTSCC45 | HNSCC |
| HpV16 E4 | RAD54L2 | 4 | 4 | 3 | 51574077 | VU147T | HNSCC |
| HpV16 E4 | RAD54L2 | 15 | 6 | 3 | 51575543 | VU147T | HNSCC |
| HpV16 L2 | PPFIA1 | 5 | 0 | 11 | 70115416 | VU147T | HNSCC |
