# Supplementary material for: Genomic characterization of human papillomavirus-positive and -negative human squamous cell cancer cell lines
Source: Oncotarget. 2017 Sep 21;8(49):86369–83. doi: 10.18632/oncotarget.21174 (PMC5689691; doi:10.18632/oncotarget.21174)
Supplement: Supplementary file 4 [file oncotarget-08-86369-s004.docx]

**Supplementary Table 5.** Genes with recurrent integration in HPV positive cell lines and TCGA tumor samples

| **Gene name** | **Protein name** | **Number of samples with integration** | **Chromosome** | **Function** |
| --- | --- | --- | --- | --- |
| *CTSE* | Cathepsin E | 2 | 1 | A1 peptidase involved in antigen processing and the maturation of secretory proteins |
| *FHAD1* | Forkhead associated phosphopeptide binding domain 1 | 2 | 1 | Unknown |
| *FLJ37453* | Uncharacterized LOC729614 | 2 | 1 | Unknown |
| *TP63* | Tumor protein 63 | 6 | 3 | Member of the p53 family of transcription factors |
| *LEPREL1* | Prolyl 3-hydroxylase 2 | 4 | 3 | Involved in collagen chain assembly, stability, and cross-linking |
| *FLJ46066* | Long intergenic non-protein coding RNA 1994 | 3 | 3 | Upstream from EIF4E pseudogene 3 |
| *CLDN1* | Claudin 1 | 2 | 3 | Integral membrane protein and a component of tight junction strands |
| *PTPN13* | Protein tyrosine phosphatase, non-receptor type 13 | 4 | 4 | Regulates a variety of cellular processes, including cell growth, differentiation, and mitosis |
| *CXCL6* | C-X-C motif chemokine ligand 6 | 2 | 4 | Chemotactic and angiogenic properties; strong antibacterial activity |
| *PVT1* | PVT-1 oncogene | 11 | 8 | Long noncoding RNA locus identified as a candidate oncogene |
| *MYC* | V-myc avian myelocytomatosis viral oncogene homolog | 7 | 8 | Activates the transcription of growth-related genes |
| *MIR1204* | MicroRNA 1204 | 3 | 8 | Unknown |
| *PSKH2* | Protein serine kinase H | 3 | 8 | Transferase activity |
| *POU5F1B* | POU class 5 homeobox 1B | 7 | 8 | Weak transcriptional activator highly similar to the POU class 5 homeobox 1 transcription factor |
| *ACTL7B* | Actin-like 7B | 3 | 9 | Involved in vesicular transport, spindle orientation, nuclear migration, and chromatin remodeling |
| *CD274* | Programmed cell death 1 ligand 1 (PD-L1) | 3 | 9 | Immune inhibitory receptor ligand; interaction of this ligand with its receptor inhibits T-cell activation and cytokine production |
| *KIAA1432* | RIC1 homolog, RAB6A GEF complex partner 1 | 2 | 9 | Participates in the recycling of mannose-6-phosphate receptors |
| *CUL2* | Cullin 2 | 2 | 10 | Component of multiple cullin-RING-based ECS (ElonginB/C-CUL2/5-SOCS-box protein) E3 ubiquitin-protein ligase complexes; mediates the ubiquitination of target proteins (HIF1α) |
| *TEAD1* | TEA domain transcription factor 1 | 4 | 11 | Ubiquitous transcriptional enhancer factor |
| *KRT5* | Keratin 5 | 2 | 12 | Epidermis development |
| *C12orf37* | Putative uncharacterized protein (LINC00615) | 2 | 12 | Unknown |
| *KLF12* | Krueppel-like factor 12 | 15 | 13 | Represses AP-2 alpha gene expression |
| *MIPOL1* | Mirror-image polydactyly gene 1 protein | 2 | 14 | Encoded protein may function as a tumor suppressor |
| *RAD51B* | RAD51 paralog B | 7 | 14 | Involved in the homologous recombination repair pathway |
| *SSTR1* | Somatostatin receptor 1 | 2 | 14 | Peptide hormone that regulates neurotransmission, cell proliferation, and endocrine signaling |
| *GRB7* | Growth factor receptor bound protein 7 | 2 | 17 | Adapter protein that interacts with receptor tyrosine kinases and signaling molecules (EGFR); promotes activation of STAT3, AKT1, MAPK1, MAPK3, and HRAS |
| *ERBB2* | Erb-b2 receptor tyrosine kinase 2 | 6 | 17 | Binds to ligand-bound EGF receptor family members, stabilizes ligand binding, and enhances kinase-mediated activation of downstream signalling pathways (MAPK, PI3K) |
| *MIEN1* | Migration and invasion enhancer 1 | 2 | 17 | Increases cell migration by inducing filopodia formation at the leading edge of migrating cells; plays a role in regulation of apoptosis, possibly through control of CASP3 |
| *MACROD2* | O-acetyl-ADP-ribose deacetylase MACROD2 | 4 | 20 | Removes ADP-ribose from glutamate residues in proteins bearing a single ADP-ribose moiety |
| *COL4A6* | Collagen type IV alpha 6 chain | 2 | 23 | One subunit of type IV collagen |
