# Supplementary material for: Genomic characterization of human papillomavirus-positive and -negative human squamous cell cancer cell lines
Source: Oncotarget. 2017 Sep 21;8(49):86369–83. doi: 10.18632/oncotarget.21174 (PMC5689691; doi:10.18632/oncotarget.21174)
Supplement: Supplementary file 5 [file oncotarget-08-86369-s005.docx]

**Supplementary Table 6.** Antibodies (Ab) used for reverse phase protein array (RPPA) analysis

| **Official Ab name** | **Ab name reported on dataset** | **Gene name** | **Supplier** | **Catalog #** |
| --- | --- | --- | --- | --- |
| 14-3-3 beta | 14-3-3-beta | YWHAB | Santa Cruz | sc-628 |
| 14-3-3 epsilon | 14-3-3-epsilon | YWHAE | Santa Cruz | sc-23957 |
| 14-3-3 zeta | 14-3-3-zeta | YWHAZ | Santa Cruz | sc-1019 |
| 4E-BP1 | 4E-BP1 | EIF4EBP1 | CST | 9452 |
| 4E-BP1 (phospho S65) | 4E-BP1_pS65 | EIF4EBP1 | CST | 9456 |
| 53BP1 | 53BP1 | TP53BP1 | CST | 4937 |
| Acetyl CoA Carboxylase (phospho S79) | ACC_pS79 | ACACA, ACACB | CST | 3661 |
| Acetyl CoA Carboxylase 1 | ACC1 | ACACA | Abcam | ab45174 |
| ADAR1 | ADAR1 | ADAR | Abcam | ab88574 |
| Akt | Akt | AKT1,2,3 | CST | 4691 |
| Akt (phospho S473) | Akt_pS473 | AKT1,2,3 | CST | 9271 |
| Akt (phospho T308) | Akt_pT308 | AKT1,2,3 | CST | 2965 |
| AMPK alpha | AMPKa | PRKAA1 | CST | 2532 |
| AMPK alpha (phospho T172) | AMPKa_pT172 | PRKAA1 | CST | 2535 |
| AMPK alpha 2 (Phospho S345) | AMPK-a2_pS345 | PRKAA2 | Abcam | ab129081 |
| Androgen Receptor | AR | AR | Abcam | ab52615 |
| Annexin I | Annexin-I | ANXA1 | BD Biosciences | 610066 |
| Annexin VII | Annexin-VII | ANXA7 | BD Biosciences | 610668 |
| A-Raf | A-Raf | ARAF | CST | 4432 |
| ARID1A | ARID1A | ARID1A | Sigma-Aldrich | HPA005456 |
| Atg3 | Atg3 | ATG3 | CST | 3415 |
| Atg7 | Atg7 | ATG7 | CST | 8558 |
| ATM | ATM | ATM | CST | 2873 |
| ATM (phospho S1981) | ATM_pS1981 | ATM | CST | 5883 |
| ATR (Phospho S428) | ATR_pS428 | ATR | Abcam | ab178407 |
| ATRX | ATRX | ATRX | Abcam | ab97508 |
| Aurora B/AIM1 | Aurora-B | AIM1 | CST | 3094 |
| Axl | Axl | AXL | CST | 8661 |
| B7-H4 | B7-H4 | VTCN1 | CST | 14572 |
| Bad (phospho S112) | Bad_pS112 | BAD | CST | 9291 |
| Bak | Bak | BAK1 | Abcam | ab32371 |
| BAP1 | BAP1 | BAP1 | Santa Cruz | sc-28383 |
| Bax | Bax | BAX | CST | 2772 |
| Bcl2 | Bcl2 | BCL2 | Dako | M0887 |
| Bcl2A1 | Bcl2A1 | BCL2A1 | Abnova | PAB8528 |
| Bcl-xL | Bcl-xL | BCL2L1 | CST | 2762 |
| Beclin | Beclin | BECN1 | Santa Cruz | sc-10086 |
| beta Actin | b-Actin | ACTB | CST | 4970 |
| beta Catenin | b-Catenin | CTNNB1 | CST | 9562 |
| beta Catenin (phospho T41/S45) | b-Catenin_pT41_S45 | CTNNB1 | CST | 9565 |
| Bid | Bid | BID | Abcam | ab32060 |
| Bim | Bim | BCL2L11 | Abcam | ab32158 |
| BiP/GRP78 | BiP-GRP78 | HSPA5 | BD Biosciences | 610978 |
| B-Raf | B-Raf | BRAF | Abcam | ab33899 |
| B-Raf (phospho S445) | B-Raf_pS445 | BRAF | CST | 2696 |
| BRD4 | BRD4 | BRD4 | CST | 13440 |
| c-Abl | c-Abl | ABL | CST | 2862 |
| c-IAP2 | c-IAP2 | BIRC3 | CST | 3130 |
| Caspase-3 active | Caspase-3 | CASP3 | Abcam | ab32042 |
| Caspase-7 (cleaved D198) | Caspase-7-cleaved | CASP7 | CST | 9491 |
| Caspase-8 | Caspase-8 | CASP8 | CST | 9746 |
| Caveolin-1 | Caveolin-1 | CAV1 | CST | 3238 |
| CD171 (L1) | CD171 | L1CAM | BioLegend | 826701 |
| CD26 | CD26 | CD26 | Abcam | ab28340 |
| CD29 | CD29 | ITGB1 | BD Biosciences | 610467 |
| CD31 | CD31 | PECAM1 | Dako | M0823 |
| CD44 | CD44 | CD44 | CST | 3570 |
| CD49b | CD49b | ITGA2 | BD Biosciences | 611016 |
| cdc2 (Phospho Y15) | cdc2_pY15 | CDK1 | CST | 4539 |
| cdc25C | cdc25C | CDC25C | CST | 4688 |
| CDK1 | CDK1 | CDK1 | Abcam | ab32384 |
| CDKN2A/p16INK4a | p16INK4a | CDKN2A | Abcam | ab81278 |
| Chk1 | Chk1 | CHEK1 | CST | 2360 |
| Chk1 (phospho S296) | Chk1_pS296 | CHEK1 | Abcam | ab79758 |
| Chk2 | Chk2 | CHEK2 | CST | 3440 |
| Chk2 (phospho T68) | Chk2_pT68 | CHEK2 | CST | 2197 |
| c-Jun ( phospho S73) | c-Jun_pS73 | JUN | CST | 9164 |
| c-Kit | c-Kit | KIT | Abcam | ab32363 |
| Claudin 7 | Claudin-7 | CLDN7 | Novus Biologicals | NB100-91714 |
| c-Met | c-Met | MET | CST | 3127 |
| c-Met (phospho Y1234/Y1235) | c-Met_pY1234_Y1235 | MET | CST | 3129 |
| c-Myc | c-Myc | MYC | Santa Cruz | sc-764 |
| COG3 | COG3 | COG3 | ProteinTech | 11130-1-AP |
| COL6A1 | Collagen-VI | COL6A1 | Santa Cruz | sc-20649 |
| Connexin 43 | Connexin-43 | CNST43 | CST | 3512 |
| Cox2 | Cox2 | PTGS2 | CST | 4842 |
| Cox-IV | Cox-IV | PTGS3 | CST | 4850 |
| C-Raf (phospho S338) | C-Raf_pS338 | RAF1 | CST | 9427 |
| C-Raf/Raf-1 | C-Raf | RAF1 | Millipore | 04-739 |
| CREB | CREB | CREB1 | CST | 9197 |
| Cyclin B1 | Cyclin-B1 | CCNB1 | Epitomics | 1495-1 |
| Cyclin D1 | Cyclin-D1 | CCND1 | Santa Cruz | sc-718 |
| Cyclin D3 | Cyclin-D3 | CCND3 | CST | 2936 |
| Cyclin E1 | Cyclin-E1 | CCNE1 | Santa Cruz | sc-247 |
| Cyclophilin F | Cyclophilin-F | PPIF | Abcam | ab110324 |
| Detyrosinated alpha-Tubulin | D-a-Tubulin | TUBA1A | Abcam | ab48389 |
| Dimethyl-Histone H3 (Lys4) | DM-Histone-H3 | HISTH3 | Millipore | 07-030 |
| Dimethyl-K9 Histone H3 | DM-K9-Histone-H3 | H3K9ME2 | Abcam | ab32521 |
| DUSP4/MKP2 | DUSP4 | DUSP4 | CST | 5149 |
| E2F-1 | E2F1 | E2F1 | Santa Cruz | sc-251 |
| E-Cadherin | E-Cadherin | CDH1 | CST | 3195 |
| eEF2 | eEF2 | EEF2 | CST | 2332 |
| eEF2K | eEF2K | EEF2K | CST | 3692 |
| EGFR | EGFR | EGFR | CST | 2232 |
| EGFR (phospho Y1173) | EGFR_pY1173 | EGFR | Abcam | ab32578 |
| eIF4E | eIF4E | EIF4E | CST | 9742 |
| eIF4E (Phospho S209) | eIF4E_pS209 | EIF4E | Abcam | ab76256 |
| eIF4G | eIF4G | EIF4G1 | CST | 2498 |
| Elk1 (phospho S383) | Elk1_pS383 | ELK1 | CST | 9181 |
| ENY2 | ENY2 | ENY2 | GeneTex | GTX629542 |
| Epithelial Membrane Antigen | EMA | EMA | Dako | M061329-2 |
| ErbB2/HER2 | HER2 | ERBB2 | Lab Vision | MS-325-P1 |
| ErbB2/HER2 (phospho Y1248) | HER2_pY1248 | ERBB2 | R&D Systems | AF1768 |
| ErbB3/HER3 | HER3 | ERBB3 | Santa Cruz | sc-285 |
| ErbB3/HER3 (phospho Y1289) | HER3_pY1289 | ERBB3 | CST | 4791 |
| ERCC1 | ERCC1 | ERCC1 | Santa Cruz | sc-17809 |
| ERCC5 | ERCC5 | ERCC5 | ProteinTech | 11331-1-AP |
| ERRFI1/MIG6 | MIG6 | ERRFI1 | Sigma-Aldrich | WH0054206M1 |
| Estrogen Receptor | ER | ESR1 | Lab Vision | RM-9101 |
| Estrogen Receptor alpha (Phospho S118) | ER-a_pS118 | ESR1 | Abcam | ab32396 |
| Ets-1 | Ets-1 | ETS1 | Bethyl | A303-501A |
| FAK | FAK | PTK2 | Abcam | ab40794 |
| FAK (phospho Y397) | FAK_pY397 | PTK2 | CST | 3283 |
| Fatty Acid Synthase | FASN | FASN | CST | 3180 |
| Fibronectin | Fibronectin | FN1 | Epitomics | 1574-1 |
| FoxM1 | FoxM1 | FOXM1 | CST | 5436 |
| FoxO3a | FoxO3a | FOXO3 | CST | 2497 |
| FoxO3a (phospho S318/S321) | FoxO3a_pS318_S321 | FOXO3 | CST | 9465 |
| FRA-1 | FRA-1 | FRA1 | Santa Cruz | sc-605 |
| G6PD | G6PD | G6PD | CST | 8866 |
| Gab2 | Gab2 | GAB2 | CST | 3239 |
| GAPDH | GAPDH | GAPDH | Life Technologies | AM4300 |
| GATA3 | GATA3 | GATA3 | BD Biosciences | 558686 |
| GCLM | GCLM | GCLM | Abcam | ab124827 |
| GCN5L2 | GCN5L2 | KAT2A | CST | 3305 |
| Glutamate Dehydrogenase1/2 | Glutamate-D1-2 | GLUD | CST | 12793 |
| Glutaminase | Glutaminase | GLS | Abcam | ab156876 |
| Glycogen Synthase | Gys | GYS1 | CST | 3886 |
| Glycogen Synthase (phospho S641) | Gys_pS641 | GYS1 | CST | 3891 |
| Granzyme B | Granzyme-B | GZMB | CST | 4275 |
| GSK-3alpha/beta | GSK-3a-b | GSK3A, GSK3B | Santa Cruz | sc-7291 |
| GSK-3alpha/beta (phospho S21/S9) | GSK-3a-b_pS21_S9 | GSK3A, GSK3B | CST | 9331 |
| H2AX (phospho S140) | H2AX_pS140 | H2AX | Pierce Biotechnology | MA1-2022 |
| Heregulin | Heregulin | NRG1 | CST | 2573 |
| HES1 | HES1 | HES1 | CST | 11988 |
| Hexokinase II | Hexokinase-II | HK2 | CST | 2867 |
| Hif-1 alpha | Hif-1-alpha | HIF1A | BD Biosciences | 610958 |
| Histone H3 | Histone-H3 | H3F3A, H3F3B | Abcam | ab1791 |
| HSP27 | HSP27 | HSP27 | CST | 2402 |
| HSP27 (phospho S82) | HSP27_pS82 | HSBP1 | CST | 2401 |
| HSP70 | HSP70 | HSP70 | CST | 4872 |
| IGF1R (phospho Y1135/Y1136) | IGF1R_pY1135_Y1136 | IGF1R | CST | 3024 |
| IGFBP2 | IGFBP2 | IGFBP2 | CST | 3922 |
| IGFRb | IGFRb | INSR | CST | 3027 |
| INPP4b | INPP4b | INPP4B | CST | 4039 |
| Insulin Receptor beta | IR-b | INSRB | CST | 3025 |
| IRF-1 | IRF-1 | IRF1 | Santa Cruz | sc-497 |
| IRS1 | IRS1 | IRS1 | Millipore | 06-248 |
| Jagged1 | Jagged1 | JAG1 | Abcam | ab109536 |
| Jak2 | Jak2 | JAK2 | CST | 3230 |
| JNK/SAPK (phospho T183/Y185) | JNK_pT183_Y185 | MAPK8 | CST | 4668 |
| JNK2 | JNK2 | MAPK9 | CST | 4672 |
| LC3A/B | LC3A-B | LC3AB | CST | 4108 |
| Lck | Lck | LCK | CST | 2752 |
| LDHA | LDHA | LDHA | CST | 3582 |
| LRP6 (phospho S1490) | LRP6_pS1490 | LRP6 | CST | 2568 |
| MAPK (phospho T202/Y204) | MAPK_pT202_Y204 | MAPK1, MAPK3 | CST | 4377 |
| Mcl 1 | Mcl-1 | MCL1 | CST | 5453 |
| MDM2 (phospho S166) | MDM2_pS166 | MDM2 | CST | 3521 |
| MEK1 | MEK1 | MAP2K1 | Abcam | ab32576 |
| MEK1 (phospho S217/S221) | MEK1_pS217_S221 | MAP2K1 MAP2K2 | CST | 9154 |
| MERIT40 (Phospho S29) | MERIT40_pS29 | BABAM1 | CST | 12110 |
| Merlin/NF2 | Merlin | NF2 | Novus Biologicals | 22710002 |
| MIF | MIF | MIF | Santa Cruz | sc-20121 |
| MMP2 | MMP2 | MMP2 | CST | 4022 |
| Mnk1 | Mnk1 | MKNK1 | CST | 2195 |
| Monocarboxylic Acid Transporter 4 | MCT4 | SLC16A4 | Millipore | AB3314P |
| MSH6 | MSH6 | MSH6 | Novus Biologicals | 22030002 |
| MSI2 | MSI2 | MSI2 | Abcam | ab76148 |
| mTOR | mTOR | MTOR | CST | 2983 |
| mTOR (phospho S2448) | mTOR_pS2448 | MTOR | CST | 2971 |
| Myosin heavy chain 11 | Myosin-11 | MYH11 | Novus Biologicals | 21370002 |
| Myosin IIa (phospho S1943) | Myosin-IIa_pS1943 | MYH9 | CST | 5026 |
| Myt1 | Myt1 | MYT1 | CST | 4282 |
| NAPSIN A | NAPSIN-A | NAPSA | Abcam | ab129189 |
| N-Cadherin | N-Cadherin | CDH2 | CST | 4061 |
| NDRG1 (phospho T346) | NDRG1_pT346 | NDRG1 | CST | 3217 |
| NDUFB4 | NDUFB4 | NDUFB4 | Abcam | ab110243 |
| NF-kappaB p65 (phospho S536) | NF-kB-p65_pS536 | RELA | CST | 3033 |
| Notch1 | Notch1 | NOTCH1 | CST | 3268 |
| Notch3 | Notch3 | NOTCH3 | Santa Cruz | sc-5593 |
| N-Ras | N-Ras | NRAS | Santa Cruz | sc-31 |
| Oct-4 | Oct-4 | OCT4 | CST | 2750 |
| p21 | p21 | CDKN1A | Santa Cruz | sc-397 |
| p27 KIP 1 | p27-Kip-1 | CDKN1B | Abcam | ab32034 |
| p27/KIP 1 (phospho T198) | p27_pT198 | CDKN1B | Abcam | ab64949 |
| p38 MAPK | p38 | MAPK14 | CST | 9212 |
| p38 MAPK (phospho T180/Y182) | p38_pT180_Y182 | MAPK14 | CST | 9211 |
| p44/42 MAPK | p44-42-MAPK | MAPK3 | CST | 4695 |
| p53 | p53 | TP53 | CST | 9282 |
| p70 S6 Kinase (phospho T389) | p70-S6K_pT389 | RPS6KB1 | CST | 9205 |
| p70/S6K1 | p70-S6K1 | RPS6KB1 | Abcam | ab32529 |
| p90RSK (phospho T573) | p90RSK_pT573 | RPS6K | CST | 9346 |
| PAI-1 | PAI-1 | SERPINE1 | BD Biosciences | 612024 |
| PAICS | PAICS | PAICS | Sigma-Aldrich | HPA035895 |
| PAK1 | PAK1 | PAK1 | CST | 2602 |
| PAK4 | PAK4 | PAK4 | CST | 3242 |
| PAR | PAR | PAR | Trevigen | 4336-BPC-100 |
| PARK7/DJ1 | DJ1 | PARK7 | Abcam | ab76008 |
| PARP-1 | PARP1 | PARP1 | Santa Cruz | sc-7150 |
| PAX8 | PAX8 | PAX8 | CST | 9857 |
| Paxillin | Paxillin | PXN | Epitomics | 1500-1 |
| P-Cadherin | P-Cadherin | CDH3 | CST | 2130 |
| PCNA | PCNA | PCNA | CST | 2586 |
| Pdcd-1L1 | Pdcd-1L1 | CD274 | Santa Cruz | sc-19090 |
| Pdcd4 | Pdcd4 | PDCD4 | Rockland | 600-401-965 |
| PDGFR beta | PDGFR-b | PDGFRB | CST | 3169 |
| PDHK1 | PDHK1 | PDHK1 | CST | 3820 |
| PDK1 | PDK1 | PDPK1 | CST | 3062 |
| PDK1 (phospho S241) | PDK1_pS241 | PDPK1 | CST | 3061 |
| PD-L1 | PD-L1 | CD274 | CST | 13684 |
| PEA-15 | PEA-15 | PEA15 | CST | 2780 |
| PED/PEA-15 (phospho S116) | PEA-15_pS116 | PEA15 | Invitrogen | 44-836G |
| PI3 Kinase p110 alpha | PI3K-p110-a | PIK3CA | CST | 4255 |
| PI3K p110 beta | PI3K-p110-b | PIK3CB | Santa Cruz | sc-376412 |
| PI3K p85 | PI3K-p85 | PIK3R1 | Millipore | 06-195 |
| PKA RI alpha | PKA-a | PRKAR1A | CST | 5675 |
| PKCalpha | PKCa | PRKCA | CST | 2056 |
| PKC beta II (phospho S660) | PKC-b-II_pS660 | PRKCA, PRKCB PRKCD, PRKCE PRKCH, PRKCQ | CST | 9371 |
| PKC delta (phospho S664) | PKC-delta_pS664 | PRKCD | Millipore | 07-875 |
| PKM2 | PKM2 | PKM2 | CST | 4053 |
| PLC gamma2 (phospho Y759) | PLC-gamma2_pY759 | PLCG2 | CST | 3874 |
| PLK1 | PLK1 | PLK1 | CST | 4513 |
| PMS2 | PMS2 | PMS2 | Novus Biologicals | 22510002 |
| PRAS40 | PRAS40 | AKT1S1 | Invitrogen | AHO1031 |
| PRAS40 (phospho T246) | PRAS40_pT246 | AKT1S1 | Life Technologies | 441100G |
| PREX1 | PREX1 | PREX1 | Abcam | ab102739 |
| Progesterone Repector | PR | PGR | Abcam | ab32085 |
| PTEN | PTEN | PTEN | CST | 9552 |
| Rab11 | Rab11 | RAB11A,B | CST | 3539 |
| Rab25 | Rab25 | RAB25 | CST | 4314 |
| Rad50 | Rad50 | RAD50 | Millipore | 05-525 |
| Rad51 | Rad51 | RAD51 | CST | 8875 |
| Raptor | Raptor | RPTOR | CST | 2280 |
| Rb | Rb | RB1 | CST | 9309 |
| Rb (phospho S807/S811) | Rb_pS807_S811 | RB1 | CST | 9308 |
| RBM15 | RBM15 | RBM15 | Novus Biologicals | 21390002 |
| Rheb | Rheb | RHEB | R&D Systems | MAB3426 |
| Rictor | Rictor | RICTOR | CST | 2114 |
| Rictor (phospho T1135) | Rictor_pT1135 | RICTOR | CST | 3806 |
| RIP | RIP | RIP | CST | 4926 |
| Rock-1 | Rock-1 | ROCK1 | Santa Cruz | sc-5560 |
| RPA32 | RPA32 | RPA32 | CST | 2208 |
| RPA32 (Phospho S4/S8) | RPA32_pS4_S8 | RPA32 | Bethyl | A300-245A |
| RSK | RSK | RPS6KA1 RPS6KA2 RPS6KA3 | CST | 9347 |
| S6 (phospho S235/S236) | S6_pS235_S236 | RPS6 | CST | 2211 |
| S6 (phospho S240/S244) | S6_pS240_S244 | RPS6 | CST | 2215 |
| S6 Ribosomal Protein | S6 | RPS6 | CST | 2317 |
| SCD | SCD | SCD | Santa Cruz | sc-58420 |
| SDHA | SDHA | SDHA | CST | 11998 |
| SF2/ASF | SF2 | SRSF1 | Invitrogen | 32-4500 |
| Shc (phospho Y317) | Shc_pY317 | SHC1 | CST | 2431 |
| SHP-2 (phospho Y542) | SHP-2_pY542 | PTPN11 | CST | 3751 |
| SLC1A5 | SLC1A5 | SLC1A5 | Sigma-Aldrich | HPA035240 |
| Smac/Diablo | Smac | DIABLO | CST | 2954 |
| Smad1 | Smad1 | SMAD1 | Abcam | ab33902 |
| Smad3 | Smad3 | SMAD3 | Abcam | ab40854 |
| Smad4 | Smad4 | SMAD4 | Santa Cruz | sc-7966 |
| Snail | Snail | SNAI1 | CST | 3895 |
| SOD1 | SOD1 | SOD1 | CST | 4266 |
| SOD2 | SOD2 | SOD2 | CST | 13141 |
| Sox2 | Sox2 | SOX2 | CST | 2748 |
| Src | Src | SRC | Millipore | 05-184 |
| Src (phospho Y527) | Src_pY527 | SRC, YES1, FYN FGR | CST | 2105 |
| Src Family (phospho Y416) | Src_pY416 | SRC, LYN, FYN LCK, YES1, HCK | CST | 2101 |
| Stat3 | Stat3 | STAT3 | CST | 4904 |
| Stat3 (phospho Y705) | Stat3_pY705 | STAT3 | CST | 9131 |
| Stat5a | Stat5a | STAT5A | Abcam | ab32043 |
| Stathmin 1 | Stathmin-1 | STMN1 | Abcam | ab52630 |
| Syk | Syk | SYK | Santa Cruz | sc-1240 |
| Tau | Tau | TAU | Millipore | 05-348 |
| TAZ | TAZ | WWTR1 | CST | 4883 |
| TFAM | TFAM | TFAM | CST | 7495 |
| TIGAR | TIGAR | C12ORF5 | Abcam | ab137573 |
| Transferrin Receptor | TFRC | TFRC | Novus Biologicals | 22500002 |
| Transglutaminase II | Transglutaminase | TGM2 | Lab Vision | MS-224-P1 |
| TRIM25 | TRIM25 | TRIM25 | Abcam | ab167154 |
| TSC1/Hamartin | TSC1 | TSC1 | CST | 4906 |
| TSC2/Tuberin (phospho T1462) | Tuberin_pT1462 | TSC2 | CST | 3617 |
| TTF1 | TTF1 | NKX2-1 | Abcam | ab76013 |
| Tuberin | Tuberin | TSC2 | Abcam | ab32554 |
| TUFM | TUFM | TUFM | Abcam | ab173300 |
| Twist | TWIST | TWIST2 | Santa Cruz | sc-81417 |
| Tyro3 | Tyro3 | TYRO3 | CST | 5585 |
| UBAC1 | UBAC1 | UBAC1 | Sigma-Aldrich | HPA005651 |
| Ubiquityl Histone H2B | Ubq-Histone-H2B | H2BFM | Millipore | 05-1312 |
| UGT1A | UGT1A | UGT1A1 | Santa Cruz | sc-271268 |
| ULK1 (phospho S757) | ULK1_pS757 | ULK1 | CST | 6888 |
| VASP | VASP | VASP | CST | 3112 |
| VDAC1/Porin | Porin | VDAC1 | Abcam | ab14734 |
| VEGF Receptor 2 | VEGFR-2 | KDR | CST | 2479 |
| VHL/EPPK1** | VHL-EPPK1 | EPPK1 | BD Biosciences | 556347 |
| Vimentin | Vimentin | VIM | Dako | M0725 |
| Wee1 | Wee1 | WEE1 | CST | 4936 |
| Wee1 (Phospho S642) | Wee1_pS642 | WEE1 | CST | 4910 |
| WIPI1 | WIPI1 | WIPI1 | CST | 12124 |
| WIPI2 | WIPI2 | WIPI2 | CST | 8567 |
| XBP1 | XBP1 | XBP1 | Santa Cruz | sc-32136 |
| XPA | XPA | XPA | Santa Cruz | sc-56813 |
| XPF | XPF | XPF | Abcam | ab3299 |
| XRCC1 | XRCC1 | XRCC1 | CST | 2735 |
| YAP | YAP | YAP1 | Santa Cruz | sc-15407 |
| YAP (phospho S127) | YAP_pS127 | YAP1 | CST | 4911 |
| YB1 (phospho S102) | YB1_pS102 | YBX1 | CST | 2900 |
| ZAP-70 | ZAP-70 | ZAP70 | CST | 2705 |
